# Novel Copper(II) Coordination Compounds Containing Pyridine Derivatives of *N*^4^-Methoxyphenyl-Thiosemicarbazones with Selective Anticancer Activity

**DOI:** 10.3390/molecules29246002

**Published:** 2024-12-19

**Authors:** Roman Rusnac, Olga Garbuz, Victor Kravtsov, Elena Melnic, Dorin Istrati, Victor Tsapkov, Donald Poirier, Aurelian Gulea

**Affiliations:** 1 Laboratory of Advanced Materials in Biopharmaceutics and Technics, Institute of Chemistry, Moldova State University, MD-2009 Chisinau, Moldova; 2 Laboratory of Systematics and Molecular Phylogenetics, Institute of Zoology, Moldova State University, MD-2028 Chisinau, Moldova; 3 Laboratory of Physical Methods of Solid State Investigation “Tadeusz Malinowski”, Institute of Applied Physics, Moldova State University, MD-2028 Chisinau, Moldova; 4 Department of Dentistry, University of Medicine and Pharmacy “Nicolae Testemitanu”, MD-2004 Chisinau, Moldova; 5 Laboratory of Medicinal Chemistry, CHU de Québec Research Center, Université Laval, Québec, QC G1V 4G2, Canada; donald.poirier@crchudequebec.ulaval.ca

**Keywords:** coordination compounds, *N*^4^*-*methoxyphenyl thiosemicarbazones, anticancer agents, molecular structure, antioxidant activity, toxicity, selectivity

## Abstract

Ten coordination compounds, [Cu(L^1^)Cl] (**C1**), [Cu(L^1^)NO_3_] (**C2**), [Cu(L^2^)Cl] (C3), [Cu(L^2^)NO_3_] (**C4**), [Cu(L^3^)Cl] (**C5**), [Cu(L^3^)NO_3_] (**C6**), [Cu(L^4^)NO_3_] (**C7**), [Cu(L^4^)Cl] (**C8**), [Cu(L^5^)Cl] (**C9**), and [Cu(L^5^)NO_3_] (**C10**), containing pyridine derivatives of *N*^4^-methoxyphenyl-thiosemicarbazones were synthesized and characterized. The molecular structure of four compounds was investigated using single crystal X-ray diffraction. Spectral analysis techniques such as FT-IR, ^1^H NMR, ^13^C NMR, elemental analysis, and molar conductivity were used for all the synthesized compounds. The tested synthesized compounds were evaluated for their anticancer activity and selectivity against a variety of cancer cell lines, including HL-60, LNCaP, MCF-7, HepG-2, K-562, HeLa, BxPC-3, RD, and MDCK normal cell line. Most compounds demonstrated selective anticancer activity superior to doxorubicin. Notably, all ligands showed high antiproliferative activity against HL-60 cells, with IC_50_ values between 0.01 and 0.06 µM and a selectivity index as high as 5000. Coordination of copper(II) with ligands **HL^1^** and **HL^3^** notably enhanced antiproliferative activity, lowering the IC_50_ to 0.03 µM. Additionally, the antioxidant activity of these compounds was assessed, revealing that all tested ligands and most coordination compounds exhibited greater antioxidant activity compared to Trolox, with some ligands showing activity up to 12.3 times higher. Toxicity studies on *Daphnia magna* indicated low toxicity for the ligands, generally less than doxorubicin, with LC_50_ values ranging from 13 to 90 µM, suggesting moderate toxicity. Conversely, the coordination complexes were more toxic, with LC_50_ values between 0.5 and 13 µM.

## 1. Introduction

Heterocyclic compounds with a pyridine ring play an important role in the biochemical processes of living organisms; a majority of medical products contain heterocyclic fragments, especially pyridine rings. Natural products such as nicotinic acid, nicotine, anabasine, pyridoxine, and castor oil contain pyridinic rings, as do synthetic products such as isoniazid, pioglitazone, lansoprazole, and sulphapyridines [1]. The class of organic compounds called thiosemicarbazones has been studied in the last decade. Hundreds of articles are reported in the specialized literature with different techniques for their synthesis: (a) condensation between aldehyde or ketone and thiosemicarbazide [2]; (b) nucleophilic addition between isothiocyanate and hydrazone [3]; (c) from carbon disulfide [4,5] and reactions with hydrazine monohydrate followed by transamination reaction with a higher amine [6]. Adding pyridin-2-yl fragments to the thiosemicarbazone skeleton makes it much more effective as an anticancer agent [7,8,9]. Since the discovery of anticancer molecules based on thiosemicarbazone fragments, some potential drugs have been evaluated [10,11,12,13]. However, the majority of these thiosemicarbazones offer insufficient selectivity [14].

The methoxy group can be found in many drugs, such as trimethoprim, phenacetin, etc., that can participate in the transmethylation mechanism [15,16]. Heterocyclic thiosemicarbazones exhibit a wide spectrum of biological activities, such as antibacterial [17,18,19,20,21,22,23,24], antiviral [25,26,27], antiparasitic [28], antifungal [29,30] antimalarial [31,32], and particularly anticancer activity [28,33,34,35,36,37], but also exert effects from a catalytic perspective [38].

**Figure 1:**
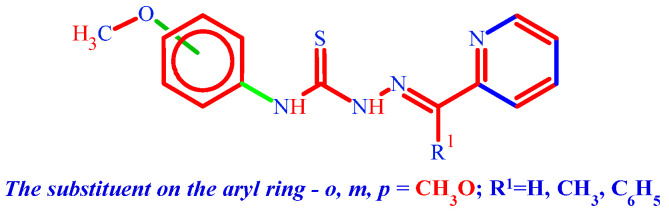
The general formula of the thiosemicarbazones **HL^1^**^–**5**^.

The aim of this study is to synthesize and characterize thiosemicarbazone compounds and to evaluate their anticancer activity and selectivity against various cancer cell lines, including HL-60, LNCaP, MCF-7, HepG-2, K-562, HeLa, BxPC-3, RD, and MDCK normal cell line. A key aspect of this research is the incorporation of the methoxy-phenyl fragment (Figure 1) into the thiosemicarbazone skeleton, which is expected to enhance membrane permeability in cancer cells and decrease the toxicity of the synthesized compounds by increasing their molecular lipophilicity.

## 2. Results and Discussion

**Scheme 1:**
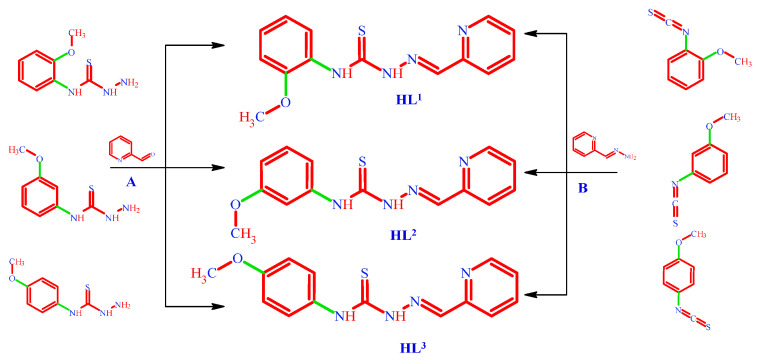
Synthesis of **HL^1^**^−**3**^ *N*-({2/or 3/or 4}methoxyphenyl)-2-[(pyridin-2-yl) methylidene] hydrazine-1-carbothioamide.

We directed our research to the synthesis of thiosemicarbazones that have the methoxyphenyl moiety substituted in the 4-position of thiosemicarbazide. We carried out the synthesis of ligands **HL^1^**^–**5**^ using two routes: method A involves the classic reaction between 2-pyridinecarboxaldehyde (Scheme 1) and the corresponding thiosemicarbazide, as described in [29,39,40].

**Scheme 2:**
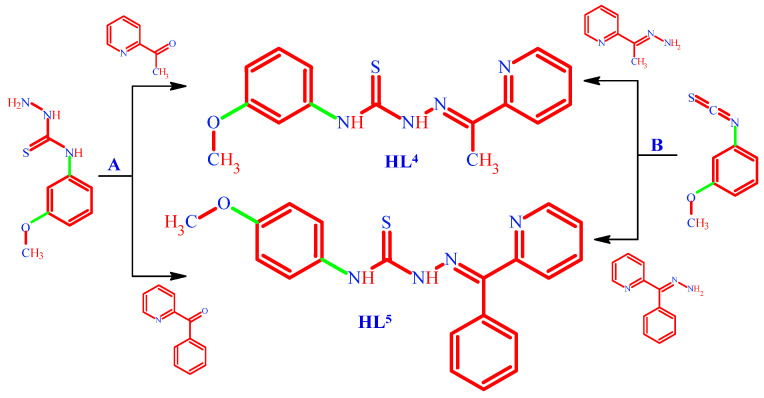
Synthesis of **HL^4^**, **HL^5^** *N*-(3-methoxyphenyl)-2-[1-(pyridin-2-yl) ethylidene] hydrazine-1-carbothioamide, and *N*-(4-methoxyphenyl)-2-[phenyl (pyridin-2-yl) methylidene]hydrazine-1-carbothioamide.

Method B is for the synthesis of thiosemicarbazones **HL^1^**^−**5**^ by the nucleophilic addition reaction between 1-isothiocyanato-2-methoxybenzene or its derivatives with hydrazones (Scheme 1 and Scheme 2). This method leads to the formation of synthesis products with high purity and quantitative yield.

The thiosemicarbazones **HL^1^**^–**5**^ were characterized by FTIR; the azomethine group >C=N [30] can be found in the IR spectrum at 1585–1570 cm^−1^, which corresponds to uncoordinated azomethine groups . The presence in the IR spectra of the ligands’ tionic group (C=S) at 1260–1245 and 830–845 [41] cm^−1^ corresponds to the uncoordinated ligand, and upon coordination with 3*d* metal ions, they lead to a shift to lower wavenumbers such as 1230–1210 cm^−1^. Absorption at 730–785 cm^−1^ in complexes **1**–**10** was assigned to stretching vibration C-S, while that at 460–510 nm was assigned to Cu(II)-thiolato vibration [42].

The ^1^H-NMR analysis [43] of **HL^1^**^−**5**^ revealed the presence of a more N-H (hydrazinic) acidic proton at 14–12 ppm [9,39,44] in the range of 3.70 to 3.80 ppm. The O-CH_3_ group stems from the structure of thiosemicarbazones **HL^1–5^**. We find the presence of N^4^H- at 8.00–8.35 ppm in the free uncoordinated **HL^1−5^** ligands. We can see the C=S group [45,46] in **HL^1−5^** ligands at 176.1–177.8 ppm in the ^13^C-NMR spectrum. Only one tautomeric tionic form is present in the solution [47,48].

### 2.1. Structure Description of ***HL^2^***, ***HL^3^***, ***HL^5^***, and Complex ***C3a***

**Figure 2:**
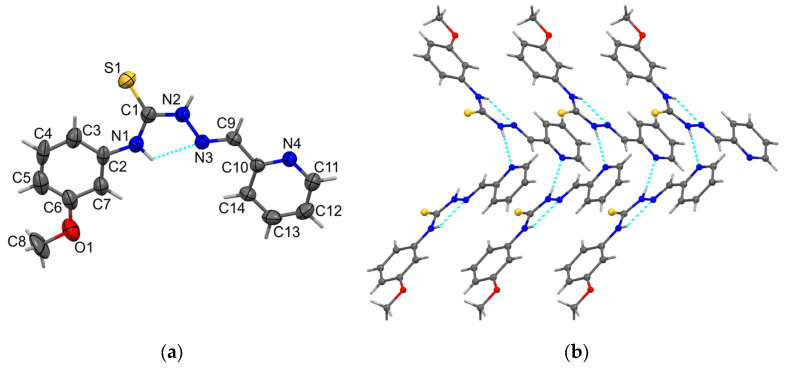
The view of molecule **HL^2^** with numbering scheme (**a**); the herringbone-like chains running along the *b* crystallographic axis in the crystals (**b**).

Crystals of **HL^2^** belong to the monoclinic space group *P*2_1_/*c*, and their structure reveals that the molecules are approximately planar with deviation of non-hydrogen atoms from their common plane within 0.07 Å while the thiosemicarbazone fragment has an E configuration (Figure 2a). The planarity of the molecule is supported by an intramolecular N1-H · N3 = 2.626 (3) Å hydrogen bond. In the crystal structure, the intermolecular N2-H · N4 = 3.043 (3) Å bonds unite the molecules in herringbone-like chains along the *b* crystallographic axis (Figure 2b)

**Figure 3:**
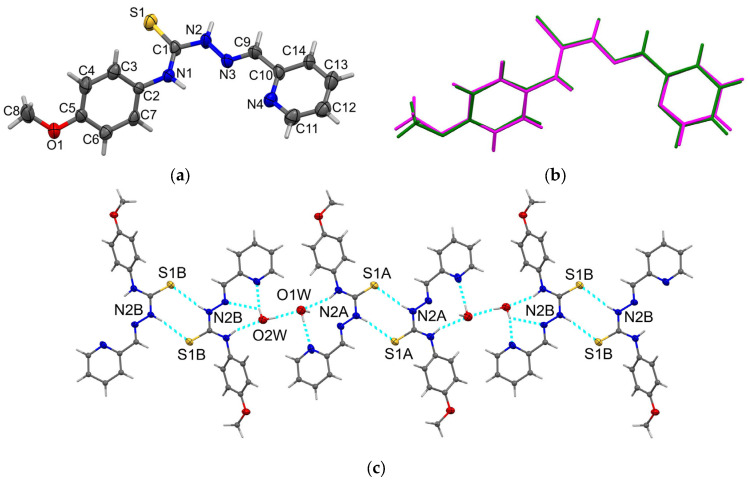
View of the **HL^3^** molecule with numbering scheme (**a**); molecular overlay in **HL^3^** (**b**); the supramolecular chain in **HL^3^** (**c**).

The crystals of **HL^3^** are separated in the form of the dehydrate **HL^3^·**2H_2_O, belonging to the centrosymmetric triclinic space group *P*-1 (Table 1, and the asymmetric unit contains two identical molecules (A and B) and two water molecules (Figure 3a). The thiosemicarbazone fragment of the molecule **HL^3^** adopts an *E* configuration, similar to **HL^2^**, but the molecule orientation of the pyridine substitution differs in **HL^2^** and **HL^3^** by the *trans*- and *cis*-position of the pyridine N4 atom with respect to the hydrazine N3 atom. The overlay of molecules A (magenta line) and B (green line) shows their similar conformations (Figure 3b). The molecules are non-planar, and the dihedral angles between the N^4^-methoxyphenyl fragment and the rest of the molecule are 46.96° and 44.86° for molecules A and B, respectively, due to rotation around single N1–C2 bonds. The corresponding torsion angles C1-N1-C2-C3 are 49.08° and 45.62° for molecules A and B, respectively.

In the crystal, each of the independent molecules A and B forms a centrosymmetric supramolecular dimer through a pair of N2A · S1A 3.373 (5) Å and N2B · S1B 3.436 (5) Å hydrogen bonds, respectively. In each dimer, H-bonds form R^2^_2_ (8) graph sets. These dimers are linked through water dimer 2 (H_2_O) by intermolecular N-H · O hydrogen bonds, forming chains in which the dimer of molecules A and the dimer of molecules B alternate with the water dimer (Figure 3c, Table S2).

**Figure 4:**
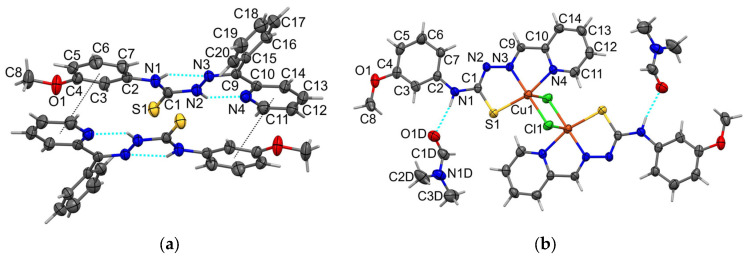
Supramolecular dimmer in **HL^5^** stabilized by intermolecular π–π stacking interactions (**a**); molecular structure of the coordination compound **C3a** (**b**).

The **HL^5^** crystallizes in the monoclinic space group *P*2_1_/*n* (Table 1). In the structure of **HL^5^**, the thiosemicarbazone fragment also adopts an E configuration, but in contrast to **HL^2^** and **HL^3^**, this ligand differs in the position of the pyridine substituent with respect to the double azomethine bond N3-C9. In **HL^2^** and **HL^3^**, the N2–N3=C9–C10 fragment has an E configuration, while in **HL^5^**, it has a Z configuration. The conformation of the molecule is supported by two intramolecular N-H · N hydrogen bonds, (Table S2) providing planarity of the molecule within (0.11 Å), except for the phenyl substituent at the C9 atom, which forms a dihedral angle of 44.56° with the rest of the molecule. The crystal structure reveals intermolecular π–π stacking interactions between center symmetry-related pyridine and anisole fragments with a Cg-Cg distance of 3.924 Å, resulting in the formation of a supramolecular dimer (Figure 4a).

Complex **C3a** crystallizes in the monoclinic space group *P*2_1_/*c* (Table 1). Structural studies revealed that **C3a** is a centrosymmetric neutral dimeric compound with the formula [Cu_2_(L^2^)_2_Cl_2_]·2DMF, in which Cl (1) atoms serve as bridging ligands (Figure 4b). Upon complexation with Cu (II), the ligand **L^2^** undergoes a configurational change of the thiosemicarbazone moiety from the *E* to the *Z* form, favoring the tridentate chelating coordination form. The Cu1 atom in the formed dimer **C3a** is pentacoordinated and the copper center exhibits a distorted square-pyramidal [49] coordination (τ = 0.158). The tridentate thiosemicarbazone ligand is coordinated to the central atom in the monodeprotonated form (**L^2^**) using an NNS set of donor atoms (Figure 4b), forming two fused five-membered CuSCNN and CuNCCN metallacycles, with the dihedral angle between their mean planes being 3.52°. The bond distances in the coordination polyhedrons Cu (1)–N (3), Cu (1)–N (4), Cu (1)–S (1), Cu (1)–Cl (1), and Cu (1)–Cl (1) * equal 1.962 (4), 2.045 (3), 2.257 (1), 2.236(1), and 2.925 (1) Å, respectively (Table S1). Two DMF solvent molecules form N1-H · O1D = 2.922 (6) Å hydrogen bonds with the dimeric complex.

### 2.2. Biological Activity

#### 2.2.1. Anticancer Activity and Selectivity

Antiproliferative activity is defined by the ability of compounds to inhibit the growth and division of cells. Compounds exhibiting antiproliferative activity may induce cell cycle arrest, promote apoptosis, or alter cell signaling pathways involved in growth regulation.

Research indicates that substances with high antioxidant activity may also possess some anticancer properties. However, this is not always the case; the presence of antioxidant activity does not guarantee that a compound will exhibit significant antiproliferative activity. For instance, some compounds may effectively neutralize free radicals but have little to no direct impact on cells. Thus, comparing antioxidant and antiproliferative activities is essential for understanding the potential therapeutic effects of various compounds. A deeper exploration of the relationship between antioxidant activity and antiproliferative activity may lead to the development of more effective drugs that can simultaneously protect cells from oxidative stress and inhibit the growth of cancer cells. 

Chemotherapy often results in severe side effects due to its cytotoxic effects on normal cells, which limits its use and may require dose reduction, interruption, or even cessation of treatment. Therefore, it is crucial for anticancer drugs to demonstrate high selectivity, targeting tumor cells specifically while sparing normal cells. 

In this study, doxorubicin and cisplatin were used as standards for comparing antiproliferative activity. Doxorubicin is recognized as one of the most potent FDA-approved chemotherapeutic agents and is a frontline drug. However, it is known for causing toxicity to most major organs, particularly cardiotoxicity, as it induces toxic damage to the mitochondria of cardiomyocytes, leading to increased oxidative stress [50,51].

Platinum-based anticancer drugs are also crucial in treating various malignant tumors, but their severe side effects, such as nephrotoxicity, have restricted their broad clinical use [52,53]. This has driven extensive research efforts and encouraged chemists to explore alternative approaches based on using endogenous metals to enhance pharmacological properties. Copper complexes are considered promising alternatives to platinum complexes as anticancer drugs due to copper’s biocompatibility and its significant roles in biological systems [53,54].

Different models like cancer cell lines, tumor explants, and enzyme systems are used to develop anticancer substances, but selection mainly relies on cancer cell lines. Assessing the antiproliferative activity of compounds serves as an initial screening approach for potential drug candidates.

In this study, we examined the antiproliferative activity of the synthesized compounds against various cell lines, including human cervical adenocarcinoma epithelial cell HeLa line, human pancreatic adenocarcinoma epithelial cell BxPC-3 line, human multinucleated rhabdomyosarcoma muscle cell RD line, human peripheral blood acute promyelocytic leukemia promyeloblast cell HL-60 line, human prostate carcinoma cell LNCaP line, human breast carcinoma cell MCF-7 line, human hepatocellular carcinoma cell HepG-2 line, human chronic myeloid leukemia bone marrow cell K-562 line, and normal canine kidney epithelial cell MDCK line. Therefore, our investigation provides valuable insights that can facilitate the preliminary screening of these substances for their anticancer activity, potentially leading to the identification of new therapeutic agents in oncology. 

The antiproliferative activity of the tested compounds was compared with the anticancer agents doxorubicin and cisplatin. The data are presented in Table 1 and Table 2. For a comparative assessment of the results, IC_50_ (μM) values were used as quantitative indicators of the efficacy of the antagonist substances in determining the optimal therapeutic dose.

The data in Table 2 indicate that doxorubicin and cisplatin exhibit high toxicity, with a low selectivity index ranging from 0.05 to 1.8. Both anticancer agents demonstrate significant anticancer activity against the studied cell lines, with doxorubicin showing greater activity compared to cisplatin. Furthermore, both substances possess antioxidant activity, with the activity of doxorubicin exceeding that of Trolox. A correlation is observed between the antioxidant activity and anticancer activity of doxorubicin (DOXO) and cisplatin (CDDP) toward the HeLa and BxPC-3 cancer cell lines.

The results regarding the antiproliferative activity of the synthesized copper(II) metal coordination compounds with thiosemicarbazone ligands **HL** and **HL^1^**^−**5**^ on HeLa, RD, and BxPC-3 cancer cell lines, as well as the MDCK normal cell line, are shown in Table 2. 

To demonstrate the selectivity of the antiproliferative activity for these cancer cell lines, the IC_50_ values for the MDCK normal cell line were used to calculate the selectivity indexes (SIs). The SIs was determined by taking the ratio of the IC_50_ value for the normal cell line to the IC_50_ value for the cancer cell line, as per Formula (1):

(1) $$SIs=\frac{{IC}_{50}\;\;\left(MDCK\right)}{\;{IC}_{50}\;\left(\;cancer\;cell\;line\right)}$$

First of all, the majority of the tested compounds demonstrated higher antiproliferative activity and selectivity compared to doxorubicin and cisplatin. The analysis of the data from this table shows that, in general, the tested compounds exhibited greater selectivity toward BxPC-3 and RD cells; for instance, the ligands **HL^1^** and **HL^3^**^−**5**^ have selectivity indices (SI) ranging from 31 to 200, whereas doxorubicin (DOXO) and cisplatin (CDDP) have SIs values from 0.1 to 2. Regarding HeLa cells, complexes **C5** and **C6**–**9** possess higher SIs values than DOXO and CDDP.

**Figure 5:**
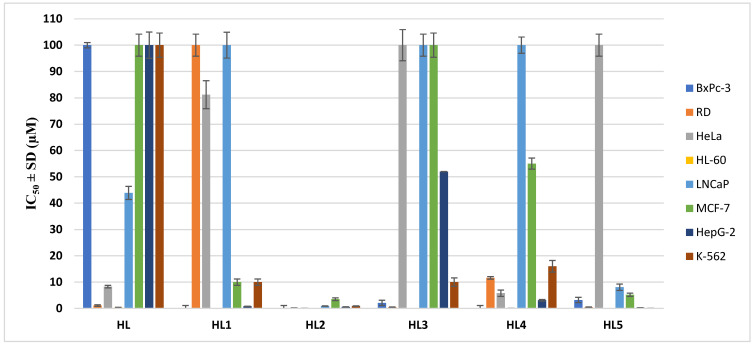
Inhibition of proliferation of HeLa, RD, BxPC-3, HL-60, LNCaP, MCF-7, HepG-2, and K-562 cancer cell lines by **HL** and **HL ^1–^**^5^. Average results of three experiments.

The thiosemicarbazones ligands **HL** and **HL^1^**^−**5**^, as shown in Figure 5, demonstrated selective antiproliferative activity against tested cancer cell lines. The ligand *N*-phenyl-2-(pyridin-2-ylmethylidene)hydrazinecarbothioamide exhibited antiproliferative activity against HeLa, RD, and BxPC-3 cell lines within the following concentration IC_50_ range: 1–100 µM. The presence of the phenyl group may enhance hydrophobic interactions, which could contribute to its overall activity. *N*-(2-Methoxyphenyl)-2-(pyridin-2-ylmethylidene) hydrazinecarbothioamide has an IC_50_ range of 0.1–100 µM. The methoxy group in the ortho position likely increases electron density on the aromatic system, enhancing its ability to improve antiproliferative effects. *N*-(3-Methoxyphenyl)-2-(pyridin-2-ylmethylidene)hydrazinecarbothioamide displays an IC_50_ range of 0.1–0.2 µM, indicating potent activity. The methoxy group at the meta position may allow for effective stabilization of the reaction intermediates and enhance the stability of the interactions ligand with cellular targets, resulting in increased potency against cancer cells. *N*-(4-Methoxyphenyl)-2-(pyridin-2-ylmethylidene) hydrazinecarbothioamide presents an IC_50_ of 0.5–100 µM. The *para*-positioned methoxy group can also enhance electron density and may contribute to favorable sterics and electronic interactions with protein targets in the cancer cells. However, its broader range of activity suggests that it might require higher concentrations for effectiveness compared to the ligands with the methoxy group in the meta and ortho positions. *N*-(3-Methoxyphenyl)-2-[1-(pyridin-2-yl) ethylidene] hydrazinecarbothioamide has an IC_50_ of 0.1–11 µM, indicating very strong activity. The presence of the ethylidene substituent increases steric hindrance, potentially facilitating better binding to specific cellular targets and improving selectivity against cancer cells. The combination of the methoxy group and this structural modification enhances its overall efficacy. *N*-(3-Methoxyphenyl)-2-[phenyl(pyridin-2-yl) methylidene] hydrazinecarbothioamide demonstrates an IC_50_ of 0.5–100 µM. The presence of both the methoxy group and the phenyl substituent may create a favorable electronic environment and steric arrangement that enhances binding to biological targets. 

The observed differences in the antiproliferative activity of these ligands can be attributed to the influence of their functional groups and the positioning of these groups within the molecules. The variation in chemical structures, particularly the presence, type, and spatial arrangement of methoxy and phenyl groups, significantly impacts their ability to interact with cancer cells. These structural features can affect solubility, permeability, and activity. Each ligand may interact with different cellular targets or pathways, leading to varying degrees of effectiveness against distinct cancer cell lines.

Coordination of the obtained pro-ligands to the 3*d* metal atoms leads to an enhancement of antiproliferative activity against HeLa, RD, and BxPC-3 cancer cell lines [55]. In many cases, the complexes even surpass doxorubicin (DOXO) and cisplatin (CDDP) in terms of activity against these three cancer cell lines. The copper complexes **C1–C4** exhibited activity within the range of IC_50_ values from 0.1 to 0.8 µM, while complexes **C6-C10** showed activity ranging from 0.07 to 3.9 µM. Notably, [Cu(L^5^)NO_3_] (**C10**) demonstrated the highest antiproliferative activity towards HeLa cells, with an IC_50_ of 70 nM.

The observed enhancement in antiproliferative activity upon coordination with 3*d* metal atoms, such as copper, can be attributed to several factors. First, the coordination of the metal ions can facilitate the stabilization of the ligand structure, making the complexes more chemically reactive and biologically active. This reactivity is essential for the interaction of these compounds with cellular components, which can lead to increased potency against cancer cells.

Second, while free ligands may exhibit certain degrees of activity, the presence of a metal center often allows for broader and more effective interactions with specific biological targets, such as DNA, proteins, or enzymes involved in cell proliferation and apoptosis. The metal ions can participate in electron transfer processes or catalytic reactions, enhancing the overall mechanism of action of the anticancer agents. Furthermore, the structural configuration and electron density around the complexes can significantly influence their ability to penetrate cell membranes and reach intracellular targets. 

The obtained results for another five cancer cell lines, namely HL-60, LNCaP, MCF-7, HepG-2, and K-562, are presented in Table 3. As in the previous case, to show the selectivity of the antiproliferative activity towards these types of cancer cells, the IC_50_ values towards the MDCK normal cell line are given alongside with IC_50_ values towards cancer cell lines, and the corresponding selectivity indexes are given. 

All investigated ligands demonstrated high antiproliferative activity against HL-60 cells, with IC_50_ values ranging from 0.01 to 0.06 µM and a high selectivity index reaching up to 5000. Coordination of copper with the proligands **HL^1,3^** significantly enhanced antiproliferative activity, reducing the IC_50_ value to 0.03 µM. In most cases, with regard to LNCaP, MCF-7, HepG-2, and K-562 cancer cell lines, the ligands exhibit selective activity, and it is often observed that copper enhances the antiproliferative activity of these cell lines.

In summary, the antiproliferative activity of the tested ligands varies significantly, with the influence of functional groups and their positions playing a crucial role in determining their effectiveness. This diversity underscores their potential for personalized therapy.

In addition, the varying IC_50_ values among the copper complexes indicate the effect of different ligands and their coordination with copper on the antiproliferative potency. Overall, the results of this study highlight the importance of metal coordination in enhancing the biological activity of ligands and suggest a promising avenue for the development of new therapeutic agents in oncology.

#### 2.2.2. Antioxidant Activity

**Figure 6:**
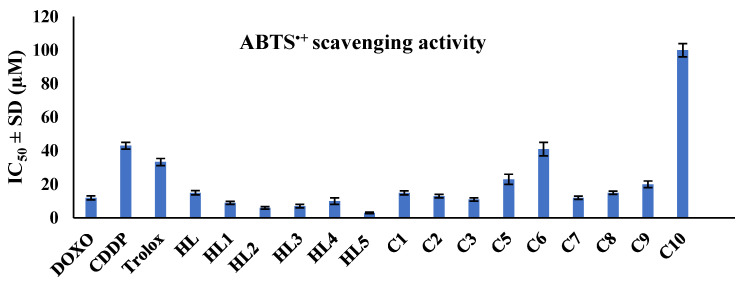
ABTS ^+^ scavenging activity of the ligands (**HL** and **HL^1−5^**), their coordination compounds (**C1**, **C2**, and **C4**–**10**), and anticancer drugs (DOXO and CDDP).

Antioxidant activity refers to the ability of compounds to neutralize free radicals and prevent oxidative damage to cells. Antioxidants can donate electrons or hydrogen atoms to free radicals, making them less reactive. This protection enables cells to defend against damage caused by oxidative stress, which is a contributor to the pathogenesis of many diseases, including cancer. Antioxidant activity is often measured using a method such as the ABTS^•+^. In our studies, the antioxidant activity of the tested compounds was measured using the ABTS^•+^ assay, with Trolox used as the standard for biochemical studies of antioxidant activity, alongside the anticancer drugs doxorubicin (DOXO) and cisplatin (CDDP) (Figure 6).

According to the presented results, the thiosemicarbazones ligands **HL^1−5^** exhibit higher antioxidant activity than the copper(II) complexes of these thiosemicarbazones **HL^1−5^**. Additionally, all ligands demonstrate higher activity in comparison with Trolox by up to 12.3 times. For ligands, the lowest antioxidant activity (IC_50_ = 14.9 µM) is observed for *N*-phenyl-2-(pyridin-2-ylmethylidene)hydrazinecarbothioamide (IUPAC name) or 2-formylpyridine *N^4^*-phenyl-thiosemicarbazide (preferred name) (HL), which may be related to its structure lacking an additional methoxy group, potentially limiting its interaction with free radicals. 

*N*-(2-Methoxyphenyl)-2-[(pyridin-2-yl) methylidene] hydrazine-1-carbothioamide (**HL^1^**) shows a significantly higher activity (IC_50_ = 9 µM) than **HL** (IC_50_ = 14.9 µM), suggesting that the methoxy group positively influences receptor binding or reaction with free radicals. The next two ligands, *N*-(3-methoxyphenyl)-2-(pyridin-2-ylmethylidene) hydrazinecarbothioamide (**HL^2^**) and *N*-(4-methoxyphenyl)-2-(pyridin-2-ylmethylidene) hydrazinecarbothioamide (**HL^3^**), with IC_50_ values of 6.2 µM and 6.7 µM, respectively, exhibit similar activity levels. This indicates that the placement of the methoxy groups at the 3 and 4 positions of the thiosemicarbazides results in similar electronic distribution and stability. This suggests that the positioning of functional groups on the phenolic ring is important for achieving high activity.

*N*-(3-Methoxyphenyl)-2-[phenyl (pyridin-2-yl) methylidene]hydrazinecarbothioamide (**HL^5^**), with an IC_50_ of 2.7 µM, demonstrates the highest antioxidant activity among the tested ligands. The methoxy group at position 3 and the phenyl group in another part of the structure may create a synergistic effect, enhancing activity through improved binding or a more effective electron transfer mechanism.

The coordination compounds of ligands **HL** and **HL^1−5^** exhibited antioxidant activity in the following sequence: [Cu(L^2^)Cl] (**C3**) ≥ [Cu(L^3^)NO_3_] (**C6**) ≥ [Cu(L^1^)NO_3_] (**C2**) ≥ [Cu(L^4^)NO_3_] (**C7**) ≥ [Cu(L^1^)Cl] (**C1**) ≥ [Cu(L^4^)Cl] (**C8**) ≥ [Cu(L^2^)NO_3_] (**C4**) ≥ [Cu(L^5^)NO_3_] (C10) ≥ [Cu(L^3^)Cl] (**C5**) ≥ [Cu(L^5^)Cl] (**C9**). The antioxidant activity of these coordination compounds can vary significantly due to their structural characteristics, electronic properties, types of ligands or anions, and the spatial arrangement within the molecule.

#### 2.2.3. In Vivo Toxicity

In vivo toxicity studies are a crucial and mandatory phase in drug development, as they allow for the assessment of safety and potential risks associated with the use of new compounds. One method for studying toxicity involves testing on model organisms such as *Daphnia magna*. This species is widely used as a model organism in ecotoxicology, providing relevant data on toxicity at the organismal level. These organisms are sensitive to a variety of chemicals, making them important tools for evaluating potential threats to both the environment and human health [56,57].

Research on *Daphnia magna* enables rapid assessment of the acute toxicity of compounds due to their short life cycle and high reproduction rate, allowing researchers to obtain results in a relatively short period of time. Testing methodologies using *Daphnia magna* are standardized and accepted by many international regulations, ensuring the comparability of results and facilitating further studies on other levels of toxicity and safety.

By studying the toxicity of new compounds in *Daphnia magna*, researchers can predict their impact on ecosystems, which is particularly significant for assessing the effects of drugs that may enter the environment through wastewater. Therefore, using *Daphnia magna* to evaluate acute toxicity is a justified and important step in the process of developing safe and effective pharmaceuticals, contributing to the reduction of potential risks to human health and the environment. 

**Figure 7:**
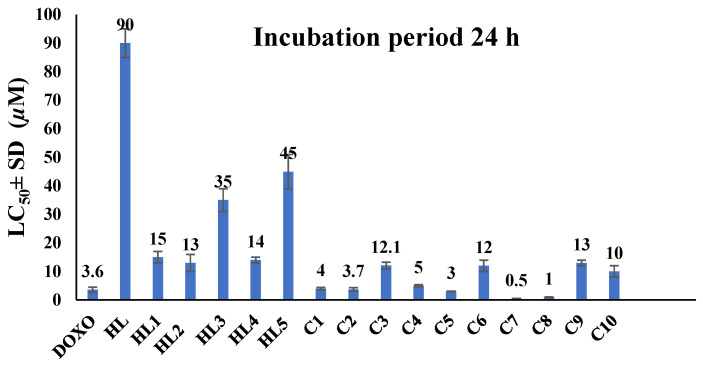
In vivo toxicity of the tested compounds and DOXO on *Daphnia magna* after 24 h incubation. The numbers indicate the median lethal concentration (LC_50_) of the tested compounds and DOXO.

In this context, the substances were tested for toxicity (Figure 7). The intensity of the influence of compounds at the median lethal concentration (LC_50_) on *Daphnia magna* was determined through microanalysis. As expected, microscopy observations of *Daphnia magna* in the control group (without the tested compounds) indicated no pathological changes in the organisms. However, after incubation with the tested compounds, *Daphnia magna* were observed at the bottom of the wells. Light microscopy revealed that many of the *Daphnia magna* were moving slowly, while others remained motionless. Additionally, deformations were noted in the limbs and trunks of the *Daphnia magna*, with their internal contents mixed with the growth media [58].

Most of the tested substances exhibited lower or compared toxicity to doxorubicin. All ligands demonstrated less toxicity than the complexes formed with these proligands. The obtained LC_50_ values for the thiosemicarbazones **HL** and **HL ^1−5^** ranged from 13 to 90 µM, indicating moderate toxicity. In contrast, the complexes showed higher toxicity, with LC_50_ values ranging from 0.5 to 13 µM. This indicates a significantly higher toxicity of the complexes compared to the proligands.

## 3. Materials and Methods

All the reagents used were chemically pure 3*d* metal salts CuCl_2_·2H_2_O, Cu(NO_3_)_2_·3H_2_O (Merck, Darmstadt, Germany), and were used as supplied. 2-Pyridinecarboxaldehyde, 2-acetylpyridine, 2-benzoylpyridine, ABTS (2,2′-azino-*bis*(3-ethylbenzothiazoline-6-sulphonic acid)), and potassium persulfate (K_2_S_2_O₈) were used as received (Merck, Germany). Reagents and solvents were used as received from commercial sources.

### 3.1. Synthesis

Tetramethylthiuram disulfide (1.5 equivalent) was added to a mixture of the respective anesidine derivatives (1.0 equivalent) and dimethylformamide (10 mL). The reaction mixture was heated and stirred at 60–75 °C for 10–18 h The resulting mixture was concentrated by rotary evaporation. The precipitate formed upon cooling was collected by vacuum filtration and chemically purified by isolating the sulfur from the reaction medium after a 5% dissolution of thiourea in the mineral acid (sulfuric acid (H_2_SO_4_), hydrochloric acid (HCl)), then neutralized with sodium bicarbonate. The precipitate formed was recrystallized from toluene. Thioures were obtained as follows: *N’*-(2-methoxyphenyl)-*N,N*-dimethylthiourea (yield 79%), *N’*-(3-methoxyphenyl)-*N,N*-dimethylthiourea (yield 87%), and *N’*-(4-methoxyphenyl)-*N,N*-dimethylthiourea (yield 95%).

The synthesis of thiosemicarbazides *N*-(2-methoxyphenyl)hydrazinecarbothioamide (yield 90%), *N*-(3-methoxyphenyl)hydrazinecarbothioamide (yield 94%), and *N*-(4-methoxyphenyl)hydrazinecarbothioamide (yield 97%) occurs upon the interaction of thioureas with hydrazine monohydrate. The nucleophilic substitution reaction is carried out in p-xylene, at reflux temperature.

When thioureas degrade with mineral acids in 1,4-dioxane, isothiocyanates are formed. The specific isothiocyanates produced include 1-isothiocyanato-2-methoxybenzene with a yield of 76%, 1-isothiocyanato-3-methoxybenzene with a yield of 67%, and 1-isothiocyanato-4-methoxybenzene with a yield of 94%.

Method A

General procedure:

For the synthesis of thiosemicarbazone **HL** by method A: one equivalent of *N*-(x-methoxyphenyl) hydrazinecarbothioamide and one equivalent of the corresponding aldehyde or ketone are taken in a synthesis flask and dissolved in absolute ethanol, adding 10 mol% of glacial acetic acid to catalyze the end condensation reaction.

Method B

Synthesis of thiosemicarbazones **HL^1−5^** requires a reaction between the corresponding isothiocyanate and hydrazone (2-[hydrazinylidenemethyl]pyridine or 2-[1-hydrazinylideneethyl] pyridine or 2-[hydrazinylidene (phenyl) methyl] pyridines) at a molar ratio of 1:1 dissolved in an aprotic solvent such as THF or 1,4-dioxane (note: it is recommended to avoid alcohols).

*N-(2-Methoxyphenyl)-2-[(pyridin-2-yl) methylidene] hydrazine-1-carbothioamide* (**HL^1^**). White crystalline solid, yield 98%; mp: 186–187 °C [59], R_f_ = 0.41 (ethyl acetate-toluene 2:1). Anal. Calc. for C_14_H_14_N_4_OS: C (58.72%) H (4.93%) N (19.57%). Found: C (58.99%) H (5.04%) N (19.21%). FW: 286.35 g·mol^−1^. ^1^H NMR (400 MHz, DMSO-*d*_6_) δ 14.47 (s, 1H, –N*H*-N), 12.10 (s, 1H, –N^4^*H*), 10.04 (d, 1H, Py), 8.61 (d, 1H, Py), 8.60 (d, 1H, Py), 8.25 (d, 1H, Py), 8.01 (s, 1H, CH=N-), 7.91–6.96 (C-H, Ar), 3.87 (s, 3H, -O–CH_3_). ^13^C NMR (101 MHz, DMSO-*d*_6_) δ 176.2 (C=S), 153.4 (HC=N), 152.7, 149.9, 143.1, 137.2, 128.0, 126.8, 125.7, 124.8, 120.5, 120.3, 111.9 (C-Ar and Py); 56.5 (-O–CH_3_). The NMR spectra (**HL^1−5^**) are provided in detail in the Supplementary Materials S1–S10. FTIR (cm^−1^): 3279, 3143 ν (N-H, hydrazinic and thioamide); 3076 ν (C-H, aryl); 2960 ν_as_ (C-H)-CH_3_); 2834 ν_sy_ (C-H)-CH_3_) and 2935 ν(C-H) aldehydes; 1600 δ (N-H/OH); 1583 ν (C=N); 1488 ν (C=C); 1235 and 836 ν (C=S); 726 *ortho*-substituted rings; (py). The IR spectra are provided in detail in the Supplementary Materials S13–S17.

*N-(3-Methoxyphenyl)-2-[(pyridin-2-yl) methylidene] hydrazine-1-carbothioamide* (**HL^2^**). White crystalline solid, yield 96%; mp: 196–197 °C, R_f_ = 0.52 (ethyl acetate-toluene 2:1).. Anal. Calc. for C_14_H_14_N_4_OS: C (58.72%) H (4.93%) N (19.57%). Found: C (58.68%) H (4.82%) N (19.87%). FW: 286.35 g·mol^−1^. ^1^H NMR (400 MHz, DMSO-*d*_6_) δ 14.45 (s, 1H, –N*H*-N), 12.09 (s, 1H, –N^4^*H*), 10.02 (d, 1H, Py), 8.60 (d, 1H, Py), 8.59 (d, 1H, Py), 7.98 (d, 1H, Py), 7.86 (s, 1H, CH=N-), 7.42–6.95 (C-H, Ar), 3.86 s, 3H, -O–CH_3_). ^13^C NMR (101 MHz, DMSO-*d*_6_) δ 176.6 (C=S); 159.5 (HC=N), 153.4, 149.8, 143.5, 140.5, 137.1, 129.3, 124.8, 121.3, 118.4, 111.9, 111.4 (C-Ar and Py); 55.6 (-O–CH_3_). (FTIR (cm^−1^): 3076 ν (C-H); 2988 ν_as_ (C-H) (sp^3^); 2833 ν_sy_ (C-H) (sp^3^); 1598 ν (C=N); 1492 ν (C=C); 741 ν (CS).

*N-(4-Methoxyphenyl)-2-[(pyridin-2-yl) methylidene] hydrazine-1-carbothioamide* (**HL^3^**). White crystalline solid, yield 97%; mp: 167–168 °C, R_f_ = 0.63 (ethyl acetate-toluene 2:1). Anal. Calc. for C_14_H_14_N_4_OS: C (58.72%) H (4.93%) N (19. Found: C (58.88%) H (4.71%) N (19.66%). FW: 286.35 g·mol^−1^. ^1^H NMR (400 MHz, DMSO-*d*_6_) δ 14.36 (s, 1H, –N*H*-N), 11.97 (s, 1H, –N^4^*H*), 10.19, 8.59, 8.58, 8.44, 8.42, 8.19, 7.87, 7.84, 7.83 (s, 1H, CH=N-), 7.41–6.94 (C-H, Ar), 3.77 (s, 3H, -O–CH_3_). ^13^C NMR (101 MHz, DMSO-*d*_6_) δ 177.2 (C=S); 157.5 (HC=N), 153.6, 149.8, 143.2, 136.9, 132.2, 128.1, 124.6, 121.1, 113.8 (C-Ar and Py); 55.7(-O–CH_3_) FTIR (cm^−1^): 3076 ν (C-H); 2988 ν_as_ (C-H) (sp^3^); 2833 ν_sy_ (C-H) (sp^3^); 1598 ν (C=N); 1492 ν (C=C); 741 ν (CS).

*N-(3-Methoxyphenyl)-2-[1-(pyridin-2-yl) ethylidene] hydrazine-1-carbothioamide* (**HL^4^**). White crystalline solid, yield 92%; mp: 137–138 °C, R_f_ = 0.39 (ethyl acetate-toluene 2:1). Anal. Calc. for C_15_H_16_N_4_OS: C (59.98%) H (5.37%) N (18.65%). Found: C (59.71%) H (5.44%) N (18.81%). FW: 300.37 g·mol^−1^. ^1^H NMR (400 MHz, DMSO-*d*_6_) δ 14.60 (s, 1H, –N*H*-N), 11.00 (s, 1H, –N^4^*H*), 10.69–6.80 (C-H, Ar and Py), 3.78 (s, 3H, -O–CH_3_), 2.48 (s, 3H, -CH_3_). ^13^C NMR (101 MHz, DMSO-*d*_6_) δ 177.47 (C=S), 159.55 (>C=N); 154.96, 149.76, 149.05, 148.9, 140.6, 136.9, 136.9, 129.2, 124.6, 121.7, 121.3, 118.5, 112.0, 111.4 (C-Ar and Py); 55.6 (-O–CH_3_); 13.0 (–CH_3_). FTIR (cm^−1^): 3076 ν (C-H); 2988 ν_as_ (C-H) (sp^3^); 2833 ν_sy_ (C-H) (sp^3^); 1598 ν (C=N); 1492 ν (C=C); 741 ν (CS).

*N-(3-Methoxyphenyl)-2-[phenyl (pyridin-2-yl) methylidene] hydrazine-1-carbothioamide* (**HL^5^**). White crystalline solid, yield 90%; mp: 189–190 °C, R_f_ = 0.55 (ethyl acetate-toluene 2:1). Anal. Calc. for C_20_H_18_N_4_OS: C (66.28%) H (5.01%) N (15.46%). Found: C (66.40%) H (4.91%) N (15.95%). FW: 362.44 g·mol^−1^. ^1^H NMR (400 MHz, DMSO-*d*_6_) δ 13.16 (s, 1H, –N*H*-N), 10.27 (s, 1H, –N^4^*H*), 8.89 (s, 1H, HC=N), 8.07–6.80 (C-H, Ar and Py), 3.76 (s, 3H, -O–CH_3_). ^13^C NMR (101 MHz, DMSO-*d*_6_) δ 176.7 (C=S), 159.5 (>C=N); 151.8, 149.3, 144.4, 140.3, 138.7, 137.1, 129.8, 129.6, 128.8, 126.7, 125.5, 118.1, 111.5, 111.5 (C-Ar and Py); 55.6 (-O–CH_3_). FTIR (cm^−1^): 3076 ν (C-H); 2988 ν_as_ (C-H) (sp^3^); 2833 ν_sy_ (C-H) (sp^3^); 1598 ν (C=N); 1492 ν (C=C); 741 ν (CS). 

### 3.2. Synthesis and Characterization of the Complexes (***C1***–***10***)

[Cu(L^1^)Cl] (**C1**)

An equimolar mixture of copper(II) chloride (CuCl_2_·2H_2_O, 0.8524 g, 5 mmol) and the ligand (1.4318 g, 5 mmol) (**C**) in ethyl alcohol was stirred at reflux for 2 h. The precipitate obtained was filtered and washed with cold ethanol, then recrystallized from ethyl alcohol. The obtained product had a dark green color. Yield: (1.7485 g, 91%). Anal. Calc. for C_14_H_13_ClCuN_4_OS: C (43.75%) H (3.41%) Cu (16.53%) N (14.58%). Found: C (44.01%) H (3.98%) Cu (16.93%) N (14.77%). FW: 384.34 g·mol^−1^. FTIR (cm^−1^): 3401 (N^4^H/OH); 3076 ν (C-H); 2988 ν_as_ (C-H) (sp^3^); 2833 ν_sy_ (C-H) (sp^3^); 1598 ν (C=N); 1492 ν (C=C); 741 ν (CS). Molar electrical conductivity (EtOH-DMSO, 9:1): 67 µS/cm. Supplementary Materials S18–S27 display detailed IR spectra for complexes **C1**–**10**. 

[Cu(L^1^)NO_3_] (**C2**) 

The synthesis of compound **2** followed the synthesis protocol for compound **1** (Cu(NO_3_)_2_·3H_2_O, 1.2080 g, 5 mmol) and the ligand (1.4318 g, 5 mmol) (**HL^1^**). The obtained product had a green color. Yield: (1.7052 g, 83%). Anal. Calc. for C_14_H_13_CuN_5_O_4_S: C (40.92%) H (3.19%) Cu (15.47%) N (17.04%). Found: C (41.02%) H (3.40%) Cu (16.07%) N (16.94%). FW: 410.89 g·mol^−1^. FTIR (cm^−1^): 3392 (N^4^H/OH); 3040 ν (C-H); 2986 ν_as_(C-H) (sp^3^); 2844 ν_sy_ (C-H) (sp^3^); 1597 ν (C=N); 1492 ν (C=C); 1335 ν_1_ (NO_3_)- monodentate, ν_1_–ν_6_ absorption bands in Supplementary Materials S18–S27 are due to the coordinated nitrate group.; 750 ν (CS). Molar electrical conductivity (EtOH-DMSO, 9:1): 52 µS/cm. 

[Cu(L^2^)Cl] (**C3**)

The synthesis of compound **3** followed the synthesis protocol for compound **1** (CuCl_2_·2H_2_O, 0.8524 g, 5 mmol) and the ligand (1.4318 g, 5 mmol) (**HL^2^**). The obtained product had a green color. Yield: (1.7295 g, 90%). Anal. Calc. for C_14_H_13_ClCuN_4_OS: C (43.75%) H (3.41%) Cu (16.53%) N (14.58%). Found: C (44.12%) H (3.71%) Cu (16.97%) N (14.59%). FW: 384.34 g·mol^−1^. FTIR (cm^−1^): 3356 (N^4^H/OH); 3081 ν (C-H); 2937 ν_as_ (C-H) (sp^3^); 2836 ν_sy_ (C-H) (sp^3^); 1565 ν (C=N); 1488 ν (C=C); 752 ν (CS). Molar electrical conductivity (EtOH-DMSO, 9:1): 61 µS/cm. Complex **C-3** was recrystallized from DMF, and as a result, the new compound [Cu_2_(L_2_)2Cl_2_]·2DMF (**C3a**) was obtained. Molar electrical conductivity of [Cu_2_(L_2_)2Cl_2_]·2DMF (C3a) (DMF): 46 µS/cm.

[Cu(L^2^)NO_3_] (**C4**)

The synthesis of compound **4** followed the synthesis protocol for compound **1** (Cu(NO_3_)_2_·3H_2_O, 1.2080 g, 5 mmol) and the ligand (1.4318 g, 5 mmol) (**HL^2^**). The obtained product had a green color. Yield: (1.5408 g, 75%). Anal. Calc. for C_14_H_13_CuN_5_O_4_S: C (40.92%) H (3.19%) Cu (15.47%) N (17.04%). Found: C (41.10%) H (3.34%) Cu (15.97%) N (17.04%). FW: 410.89 g·mol^−1^. FTIR (cm^−1^): 3405 (N^4^H/OH); 3083 ν (C-H); 2995 ν_as_ (C-H) (sp^3^); 2867 ν_sy_ (C-H) (sp^3^); 1596 ν (C=N); 1483 ν (C=C); 1332 ν_1_ (NO_3_)-monodentate; 740 ν (CS). Molar electrical conductivity (EtOH-DMSO, 9:1): 49 µS/cm.

[Cu(L^3^)Cl] (**C5**)

The synthesis of compound **5** followed the synthesis protocol for compound **1** (CuCl_2_·2H_2_O, 0.8524 g, 5 mmol) and the ligand (1.4318 g, 5 mmol) (**HL^3^**). The obtained product had a green color. Yield: (1.7487 g, 91%). Anal. Calc. for C_14_H_13_ClCuN_4_OS: C (43.75%) H (3.41%) Cu (16.53%) N (14.58%). Found: C (44.20%) H (3.49%) Cu (16.66%) N (14.77%). FW: 384.34 g·mol^−1^. FTIR (cm^−1^): 3458 (N^4^H/OH); 3043 ν (C-H); 2940 ν_as_(C-H) (sp^3^); 2856 ν_sy_ (C-H) (sp^3^); 1580 ν (C=N); 1494 ν (C=C); 743 ν (CS). Molar electrical conductivity (EtOH-DMSO, 9:1): 55 µS/cm. 

[Cu(L^3^)NO_3_] (**C6**)

The synthesis of compound **6** followed the synthesis protocol for compound **1** (Cu(NO_3_)_2_·3H_2_O, 1.2080 g, 5 mmol) and the ligand (1.4318 g, 5 mmol) (**HL^3^**). The obtained product had a green color. Yield: (1.7668 g, 86%). Anal. Calc. for C_14_H_13_CuN_5_O_4_S: C (40.92%) H (3.19%) Cu (15.47%) N (17.04%). Found: C (41.19%) H (3.41%) Cu (16.18%) N(16.98%). FW: 410.89 g·mol^−1^. FTIR (cm^−1^): 3321 (N^4^H/OH); 3059 ν (C-H); 2987 ν_as_ (C-H) (sp^3^); 2907 ν_sy_ (C-H) (sp^3^); 1596 ν (C=N); 1488 ν (C=C); 1305 ν_1_ (NO_3_)-monodentate; 738 ν (CS). Molar electrical conductivity (EtOH-DMSO, 9:1): 64 µS/cm. 

[Cu(L^4^)NO_3_] (**C7**)

The synthesis of compound **7** followed the synthesis protocol for compound **1** (Cu(NO_3_)_2_·3H_2_O, 1.2080 g, 5 mmol) and the ligand (1.5019 g, 5 mmol) (**HL^4^**). The obtained product had a green color. Yield: (1.8908 g, 89%). Anal. Calc. for C_15_H_15_CuN_5_O_4_S: C (42.40%) H (3.56%) Cu (14.95%) N (16.48%). Found: C (42.56%) H (3.71%) Cu (15.01%) N (16.71%). FW: 424.92 g·mol^−1^. FTIR (cm^−1^): 3322 (N^4^H/OH); 3070 ν (C-H); 2988, 2930 ν_as_ (C-H) (sp^3^); 2901, 2833 ν_sy_(C-H) (sp^3^); 1546 ν (C=N); 1483 ν (C=C); 1329 ν_1_ (NO_3_)-monodentate; 739 ν (CS). Molar electrical conductivity (EtOH-DMSO, 9:1): 70 µS/cm. 

[Cu(L^4^)Cl] (**C8**)

The synthesis of compound **8** followed the synthesis protocol for compound **1** (CuCl_2_·2H_2_O, 0.8524 g, 5 mmol) and the ligand (1.5019 g, 5 mmol) (**HL^4^**). The obtained product had a green color. Yield: (1.6332 g, 82%). Anal. Calc. for C_15_H_15_ClCuN_4_OS: C (45.22%) H (3.80%) Cu (15.95%) N (14.06%). Found: C (45.35%) H (3.98%) Cu (16.09%) N (13.96%). FW: 398.36 g·mol^−1^. FTIR (cm^−1^): 3322 (N^4^H/OH); 3072 ν (C-H); 2987 ν_as_ (C-H) (sp^3^); 2901 ν_sy_ (C-H) (sp^3^); 1565 ν (C=N); 1498 ν (C=C); 740 ν (CS). Molar electrical conductivity (EtOH-DMSO, 9:1): 58 µS/cm. 

[Cu(L^5^)Cl] (**C9**) 

The synthesis of compound **9** followed the synthesis protocol for compound **1** (CuCl_2_·2H_2_O, 0.8524 g, 5 mmol) and the ligand (1.8123 g, 5 mmol) (**HL^5^**). The obtained product had a green color. Yield: (2.2100 g, 96%). Anal. Calc. for C_20_H_17_ClCuN_4_OS: C (52.17%) H (3.72%) Cu (13.80%) N (12.17%). Found: C (52.17%) H (3.72%) Cu (13.80%) N (12.17%). FW: 460.43 g·mol^−1^. FTIR (cm^−1^): 3333 (N^4^H/OH); 3080 ν (C-H); 2980 ν_as_ (C-H) (sp^3^); 2911 ν_sy_ (C-H) (sp^3^); 1551 ν (C=N); 1490 ν (C=C); 784 ν (CS). Molar electrical conductivity (EtOH-DMSO, 9:1): 65 µS/cm. 

[Cu(L^5^)NO_3_] (**C10**) 

The synthesis of compound **10** followed the synthesis protocol for compound **1** (Cu(NO_3_)_2_·3H_2_O, 1.2080 g, 5 mmol) and the ligand (1.8123 g, 5 mmol) (**HL^5^**). The obtained product had a green color. Yield: (2.3376 g, 96%). Anal. Calc. for C_20_H_17_CuN_5_O_4_S: C (49.33%) H (3.52%) Cu (13.05%) N (14.38%). Found: C (49.51%) H (3.81%) Cu (12.98%) N (14.94%). FW: 486.99 g·mol^−1^. FTIR (cm^−1^): 3305 (N^4^H/OH); 3099, 3058 ν (C-H); 2987 ν_as_ (C-H) (sp^3^); 2936 ν_sy_ (C-H) (sp^3^); 1541 ν (C=N); 1485 ν (C=C); 1336 ν_1_ (NO_3_)- monodentate; 783 ν (CS). Molar electrical conductivity (EtOH-DMSO, 9:1): 68 µS/cm.

### 3.3. FT-IR Spectroscopy

FT-IR spectra were obtained at room temperature using a BRUKER ALPHA spectrometer (Billerica, MA, USA) over a wavelength range of 4000–400 cm⁻^1^, measured in the “Advanced Materials in Biopharmaceutics and Technics” laboratory, via OPUS software v7.5.

### 3.4. NMR Spectroscopy

^1^H and ^13^C NMR spectra were recorded with a BRUKER DRX-400 spectrometer. Chemical shifts were referenced to TMS, with DMSO-*d*_6_ as the solvent. Data were processed using MestReNova v14.1.2.

### 3.5. Molar Conductivity

Conductometric analysis was conducted using an ADWA AD8000 m (Szeged, Hungary) with calibrated electrodes. Samples were dissolved in H_2_O, DMF, DMSO, or EtOH at a concentration of 1 × 10⁻^3^ M [60,61].

### 3.6. Melting Point

The melting point was measured with a Stuart^®^ SMP10 Apparatus (Cole-Parmer Ltd., Staffordshire, UK) from ambient t emperature to 300 °C. Samples were prepared in capillaries with a layer height of 4–6 mm. 

### 3.7. Thin Layer Chromatography

TLC analysis used Macherey-Nagel plates (Düren, Germany) with a 0.2 mm Silica gel 60 layer with UV254 indicator (Düren, Germany), exploiting partition coefficient differences for separation [62].

### 3.8. X-Ray Crystallography

The crystal structures of the molecules **HL^2^**, **HL^3^**, and **HL^5^** and the complex [Cu_2_(L^2^)_2_Cl_2_]**·**2DMF **C3a**. were analyzed using single-crystal X-ray diffraction. Crystals suitable for the diffraction analysis of the organic compounds were directly selected from the synthesis process, while the crystals of **C3a** were obtained through the recrystallization of **C3** in a DMF solution.

X-ray diffraction measurements were conducted with an Xcalibur E diffractometer (Oxford Diffraction, Oxford, UK), which features a charge-coupled device (CCD) area detector and a graphite monochromator, utilizing MoKα radiation (0.71073 Å) at room temperature (293 K). The data collection, reduction, and unit cell determination were performed using the CrysAlis PRO CCD software (Oxford Diffraction Ltd., Abingdon, UK, version 1.171.33.66). The SHELXS97 and SHELXL2014/2016 software packages [63,64] were employed to solve and refine the obtained structures. The structures were solved using direct methods and refined through full-matrix least-squares on F^2^, applying anisotropic displacement parameters for all non-hydrogen atoms. Carbon-bound hydrogen atoms were positioned geometrically and treated as riding atoms, using default parameters in SHELXL (U_iso_(H) = 1.2U_eq_(C) and U_iso_(H) = 1.5U_eq_(CH_3_)), while nitrogen-bound hydrogen atoms were determined from difference Fourier maps. Summary details regarding the X-ray data and refinements for **HL^2^**, **HL^3^**, **HL^5^**, and complex **C3a** are provided in Table 3. Selected bond distances and angles for **HL^2^**, **HL^3^**, **HL^5^**, and complex **C3a** are listed in Table S1, while hydrogen bonding parameters can be found in Table S2. Visual representations of the structures were created using MERCURY software version number Mercury 2024.1.0 (Build 401958), 2024, Cambridge, UK [65].

### 3.9. Cell Proliferation Resazurin Assay

The proliferation of HeLa, BxPC-3, RD, and MDCK cells was assessed using resazurin. Triplicate cultures of 1 × 10^4^ cells in 100 μL of medium were incubated at 37 °C, 3% CO_2_, in 96-well plates. Compounds were dissolved in DMSO to form 1 × 10⁻^2^ M solutions, then diluted with media and added to wells for a 24 h incubation. Following this, 20 μL of resazurin solution was added and incubated for 4 h. Absorbance was measured at 570 nm and 600 nm.

Percentage inhibition was calculated as follows:

(2) $$I\;(\%)=100-\frac{{{Abs}_{570\;nm}}_{\left(sample\right)}-{{Abs}_{600\;nm}}_{\left(sample\right)}\;\;}{{{Abs}_{540\;nm}}_{\left(control\right)}-{{Abs}_{600\;nm}}_{\left(control\right)}\;}\times 100\;\;\;\;\;\;\;\;$$

The formula accounts for absorbance differences at 570 nm and 600 nm to determine cell viability.

### 3.10. Cell Proliferation MTT Assay

The MTT assay, a colorimetric method, quantifies viable cells to evaluate cytotoxic effects on LNCaP, MCF-7, HepG-2, and K-562 cell lines. It measures the conversion of MTT to insoluble formazan by mitochondrial succinate dehydrogenase in active cells, reflecting cell viability. Compounds were dissolved in saline and DMSO (≤0.1%) and tested at 0.1, 1, and 10 μM concentrations, following Mosmann’s method [66].

Suspension cells were centrifuged, and adherent cells were trypsinized then diluted to 5 × 10⁴ cells/mL in RPMI 1640 with 10% fetal bovine serum. Subsequently, 5 × 10^3^ cells in 100 μL were plated in 96-well plates and incubated for attachment. After adding compounds, cells were incubated for 24 h at 37 °C and 3% CO_2_. MTT reagent was introduced, and cells were monitored for formazan presence under a microscope.

Upon observing purple precipitate, detergent reagent was added, and plates were kept in the dark for 4 h. Absorbance was measured at 540 nm using a BioTek reader (Agilent, Santa Clara, CA, USA). Results, indicating percent inhibition, were calculated using the following formula:

(3) $$I\;(\%)=100-\frac{{{Abs}_{540\;nm}}_{\left(sample\right)}\;\;\;}{{{Abs}_{540\;nm}}_{\left(control\right)}\;}\times 100\;\;\;\;\;\;\;\;\;\;\;\;\;\;\;\;\;\;\;\;$$

IC_50_ values were determined as a measure of compound efficacy on cancer cell proliferation.

### 3.11. ABTS^•+^ Radical Cation Scavenging Assay

The ABTS^•+^ radical cation scavenging assay assesses antioxidant capacity by measuring the decrease in absorbance as antioxidants neutralize the ABTS^•+^ radical cation. Following Re et al.’s [67] protocol with modifications, a 7 mM ABTS solution reacted with a 2.45 mM potassium persulfate solution at 25 °C in the dark for 12–20 h to generate the radicals. The solution was diluted to an absorbance of 0.70 ± 0.01 at 734 nm.

Test compounds in DMSO were prepared at concentrations of 1 to 100 μM. In a 96-well plate, 20 μL of each dilution was combined with 180 μL of the ABTS^•+^ solution. After a 30 min incubation at 25 °C, absorbance was recorded at 734 nm using a BioTek hybrid reader. All tests were conducted in triplicate, with DMSO as the negative control. Inhibition percentage (*I*%) was calculated using the following formula:

(4) $$I\left(\%\right)=\frac{{{Abs}_{734\;nm}}_{0}-{{\;Abs}_{734\;nm}}_{1}}{{{Abs}_{734\;nm}}_{0}}\times 100\;\;$$

where Abs_0_ is the control absorbance and Abs_1_ is the absorbance with samples. This method efficiently evaluates the antioxidant capacity of both synthetic and natural compounds, aiding in the discovery of potential health-promoting substances.

### 3.12. Acute Toxicity Assay Against Daphnia magna

Toxicity testing for certain substances was conducted using *Daphnia magna*, following ISO 6341:2012 guidelines [68,69]. *Daphnia magna* were cultured parthenogenetically at the Institute of Zoology and fed *Chlorella vulgaris* grown aseptically. The algal medium included CaCl_2_, NaHCO_3_, KCl, and MgSO_4_·7H_2_O, with pH adjusted to 7.0 and O_2_ ≥ 6.00 mg/L. Juvenile daphnids were cultured in 24-well plates, each holding 10 daphnids in 1000 μL of each compound dilution. The bioassay tested concentrations from 0.1 to 100 μM to determine LC_50_, using doxorubicin as the positive control and a 0.1% DMSO solution as the negative control. The daphnids were incubated at 22 ± 2 °C with a 16 h/8 h light/dark cycle.

Mobility was assessed after 24 h; daphnids were considered immobilized if they did not swim within 15 s after gentle agitation. The experiment was conducted in triplicate to ensure accuracy.

### 3.13. Statistical Analysis

The proliferation assay results were shown as the percentage of inhibition of the tested and control compounds. The half maximal inhibitory concentration (IC_50_) indicated the effectiveness of the compounds on cell lines, as defined by FDA guidelines as the concentration needed for 50% inhibition in vitro. Toxicity was assessed via the median lethal concentration (LC_50_), the concentration causing death in 50% of juvenile daphnids, calculated using a dose–response equation via least squares fitting. All data are presented as mean ± standard deviation (SD). Statistical analyses were conducted with specialized software.

## 4. Conclusions

The novel *N*^4^-methoxyphenyl-thiosemicarbazones of pyridine derivatives **HL^1−5^** were prepared, and their structures were confirmed using various techniques, such as FTIR and NMR. **HL^2^**, **HL^3^**, **HL^5^** and [Cu_2_(L^2^)_2_Cl_2_]·2DMF **C3a** were studied by single crystal X-ray analysis. In the DMSO solution, the majority of **HL^1−5^** ligands exhibit thioamide tautomerism. All coordination compounds, **C1**–**10**, exhibit 1:1 electrolyte behavior as demonstrated by their molar conductivity measurements.

The synthesized compounds were tested against a wide spectrum of cancer cell lines, including LNCaP, MCF-7, HepG-2, K-562, HeLa, BxPC-3, and RD, along with MDCK normal cells, to evaluate their antiproliferative activity and selectivity. Most substances showed significant selective anticancer activity, exceeding that of doxorubicin and cisplatin. The complexes exhibited higher anticancer activity compared to the ligands, with the highest selectivity observed in the complexes. All ligands demonstrated high antiproliferative activity against HL-60 cells, with IC_50_ values ranging from 0.01 to 0.06 µM and a selectivity index reaching up to 5000. The coordination of copper(II) with ligands **HL^1^** and **HL^3^** significantly enhanced antiproliferative activity, reducing the IC_50_ to 0.03 µM.

Additionally, the antioxidant activity of these compounds was evaluated, showing that all tested ligands and most coordination compounds demonstrated superior antioxidant activity compared to Trolox, with some ligands exhibiting activity up to 12.3 times higher. In vivo toxicity studies on *Daphnia magna* indicated that the ligands displayed low toxicity, with LC_50_ values ranging from 13 to 90 µM, often lower than doxorubicin, suggesting moderate toxicity. On the other hand, the coordination complexes were more toxic, with LC_50_ values between 0.5 and 13 µM. These findings underscore the potential of these compounds as effective and safer alternatives in cancer therapy.

## Figures and Tables

**Table 1:** Crystal and structure refinement data for **HL^2^**, **HL^3^**, **HL^5^,** and complex **C3a**.

Compound	HL^2^	HL^3^	HL^5^	C3a
CCDC	2400994	2400995	2400996	2400997
Empirical formula	C_14_H_14_N_4_O_1_S_1_	C_14_H_16_N_4_O_2_S_1_	C_20_H_18_N_4_O_1_S_1_	C_34_H_40_Cl_2_Cu_2_N_10_O_4_S_2_
Formula weight	286.35	304.37	362.44	914.86
Crystal system	monoclinic	triclinic	monoclinic	monoclinic
Space group	*P*2_1_/*c*	*P-*1	*P*2_1_/*n*	*P*2_1_/*c*
*Unit cell dimensions*				
*a* (Å)	14.6756 (16)	9.5549 (18)	9.7269 (6)	7.1034 (5)
*b* (Å)	5.3717 (5)	12.804 (3)	16.9246 (8)	23.3341 (19)
*c* (Å)	18.506 (2)	14.078 (3)	11.1893 (6)	12.2189 (10)
α (°)	90	109.966 (18)	90	90
β (°)	102.331 (10)	97.625 (15)	96.043 (5)	102.395 (7)
γ (°)	90	108.195 (19)	90	90.895 (7)
*V* (Å^3^)	1425.2 (3)	1481.6 (6)	1831.8 (2)	90
*Z*	4	4	4	2
ρ_calc_ (g cm^−3^)	1.335	1.365	1.314	1.536
μ_Mo_ (mm^−1^)	0.228	0.229	0.193	0.928
*F*(000)	600	640	760	940
Crystal size (mm)	0.80 × 0.05 × 0.03	0.15 × 0.07 × 0.05	0.60 × 0.17 × 0.08	0.60 × 0.10 × 0.03
θ Range (°)	3.228–25.05	2.968–24.499	2.932–25.044	3.063–25.05
Index range	–17 ≤ *h* ≤ 10,	–11 ≤ *h* ≤ 11	–11≤ *h* ≤ 11	–8 ≤ *h* ≤ 8
	–6 ≤ *k* ≤ 3,	–14 ≤ *k* ≤ 14	–20 ≤ *k* ≤ 19	–16 ≤ *k* ≤ 27
	–16 ≤ *l* ≤ 22	–16 ≤ *l* ≤ 142	–13 ≤ *l* ≤ 6	–14 ≤ *l* ≤ 14
Reflections collected/unique	2519/2519 (*R*_int_ = 0.0297)	8287/4909 (*R*_int_ = 0.0699)	5970/3238 (*R*_int_ = 0.0236)	10,960/3506 (*R*_int_ = 0.0672)
Reflections with *I* > 2σ(*I*)	1351	1800	2230	2636
Number of refined parameters	182	393	236	247
Goodness-of-fit (GOF)	0.998	1.000	1.000	1.003
*R* (for *I* > 2σ(*I*))	*R*_1_ = 0.0561 *wR*_2_ = 0.1001	*R*_1_ = 0.0860 *wR*_2_ = 0.1240	*R*_1_ = 0.0463 *wR*_2_ = 0.1081	*R*_1_ = 0.0629 *wR*_2_ = 0.1668
*R* (for all reflections)	*R*_1_ = 0.1227 *wR*_2_ = 0.1242	*R*_1_ = 0.2230 *wR*_2_ = 0.1610	*R*_1_ = 0.0763 *wR*_2_ = 0.1236	*R*_1_ = 0.0864 *wR*_2_ = 0.1897
Δρ_max_/Δρ_min_ (*e*·Å^−3^)	0.206/−0.181	0.499/−0.266	0.179/−0.199	0.922/−0.515

**Table 2:** Antiproliferative activity and selectivity of the tested compounds against HeLa, RD, and BxPC-3 cancer cell lines, as well as the MDCK normal cell line.

Compound	MDCK	BxPc-3		RD		HeLa	
	IC_50_, µM	IC_50_, µM	SI	IC_50_, µM	SI	IC_50_, µM	SI
DOXO	10.8 ± 1.2	6.0 ± 0.1	1.8	16.2 ± 1.4	0.7	6.2 ± 0.5	1.7
CDDP	1.5 ± 0.3	11.2 ± 1.3	0.1	4.6 ± 0.6	0.3	30.9 ± 1.2	0.04
**HL**	100.0 ± 3.5	100.0 ± 3.0	1	1.1 ± 0.3	90.9	8.3 ± 0.5	12.0
**HL^1^**	10.1 ± 1.2	0.10 ± 0.05	101	100.0 ± 4.2	0.1	81.2 ± 5.3	0.1
**HL^2^**	0.40 ± 0.06	0.10 ± 0.04	4	0.2 ± 0.03	2.0	0.10 ± 0.01	4.0
**HL^3^**	100.0 ± 4.6	2.1 ± 0.2	48	0.5 ± 0.04	200	100.0 ± 5.9	1.0
**HL^4^**	14.9 ± 1.6	0.10 ± 0.03	149	11.6 ± 0.5	1.3	5.8 ± 1.2	2.6
**HL^5^**	100.0 ± 3.8	3.2 ± 0.3	31	0.5 ± 0.06	200	100.0 ± 4.2	1.0
[Cu(L^1^)Cl] (**C1**)	0.30 ± 0.02	0.10 ± 0.02	3	0.10 ± 0.01	3.0	0.80 ± 0.04	0.4
[Cu(L^1^)NO_3_] (**C2**)	0.60 ± 0.03	0.10 ± 0.01	6	0.10 ± 0.01	6.0	0.80 ± 0.03	0.8
[Cu(L^2^)NO_3_] (**C4**)	1.3 ± 0.4	0.10 ± 0.05	13	0.10 ± 0.06	13	0.10 ± 0.01	13.0
[Cu(L^3^)Cl] (**C5**)	26.6 ± 2.4	9.4 ± 1.2	3	19.7 ± 2.4	1.4	26.1 ± 1.2	1.0
[Cu(L^3^)NO_3_] (**C6**)	2.1 ± 1.5	0.10 ± 0.04	19	1.20 ± 0.03	1.8	1.10 ± 0.05	1.9
[Cu(L^4^)NO_3_] (**C7**)	0.90 ± 0.04	0.20 ± 0.05	5	0.50 ± 0.06	1.8	0.10 ± 0.04	9.0
[Cu(L^4^)Cl] (**C8**)	1.10 ± 0.06	0.10 ± 0.07	11	0.20 ± 0.04	5.5	0.10 ± 0.03	11.0
[Cu(L^5^)Cl] (**C9**)	13.1 ± 1.2	0.10 ± 0.03	131	0.20 ± 0.04	65.5	3.9 ± 0.2	3.4
[Cu(L^5^)NO_3_] (**C10**)	0.20 ± 0.04	0.20 ± 0.04	1	0.30 ± 0.05	0.7	0.070 ± 0.004	2.9

Note: **HL**: *N*-phenyl-2-[(pyridin-2-yl) methylidene] hydrazine-1-carbothioamide.

**Table 3:** Antiproliferative activity and selectivity of the tested compounds against HL-60, LNCaP, MCF-7, HepG-2, and K-562 cancer cell lines.

Compound	HL-60		LNCaP		MCF-7		HepG-2		K-562	
	IC_50_, µM	SI	IC_50_, µM	SI	IC_50_, µM	SI	IC_50_, µM	SI	IC_50_, µM	SI
DOXO	1.0 ± 0.3	11	1.10 ± 0.02	0.10	14.0 ± 1.5	0.80	2.8 ± 1.1	3.9	0.40 ± 0.03	27
**HL**	0.040 ± 0.005	2500	43.9 ± 2.5	0.40	100.0 ± 4.2	1.00	100.0 ± 5.0	1.0	100.0 ± 4.6	1
**HL^1^**	0.040 ± 0.006	253	100.0 ± 4.9	9.9	10.0 ± 1.2	1.00	0.80 ± 0.04	12.6	10.0 ± 1.2	1
**HL^2^**	0.010 ± 0.004	40	0.90 ± 0.05	2.3	3.5 ± 0.5	0.10	0.60 ± 0.06	0.7	0.90 ± 0.05	0.4
**HL^3^**	0.010 ± 0.001	10,000	100.0 ± 4.2	1.00	100.0 ± 4.6	1.0	52.00 ± 0.01	1.9	10.0 ± 1.6	10
**HL^4^**	0.060 ± 0.004	248	100.0 ± 3.1	6.7	55.0 ± 2.1	0.30	3.1 ± 0.3	4.8	16.0 ± 2.2	0.9
**HL^5^**	0.020 ± 0.006	5000	8.1 ± 1.2	0.10	5.2 ± 0.6	19.2	0.30 ± 0.01	333.3	0.10 ± 0.01	1000
[Cu(L^1^)Cl] (**C1**)	35.0 ± 2.1	0.010	45.0 ± 2.1	150	100.0 ± 4.2	0.003	68.0 ± 4.2	0.04	64.0 ± 1.4	0.005
[Cu(L^1^)NO_3_] (**C2**)	0.030 ± 0.001	20	0.8 ± 0.03	1.3	0.10 ± 0.05	6.0	0.40 ± 0.05	1.5	0.50 ± 0.01	1.2
[Cu(L^3^)NO_3_] (**C6**)	0.030 ± 0.006	70	0.3 ± 0.04	0.10	0.60 ± 0.03	3.5	0.60 ± 0.03	3.5	0.030 ± 0.002	70
[Cu(L^5^)Cl] (C9)	0.20 ± 0.02	66	10.0 ± 1.2	0.80	9.4 ± 1.2	1.40	14.0 ± 1.2	0.9	19.0 ± 1.5	0.7
[Cu(L^5^)NO_3_] (**C10**)	10.0 ± 1.2	0.020	1.00 ± 0.06	5.0	0.10 ± 0.01	2.0	16.0 ± 2.4	0.01	10.0 ± 3.1	0.02

Note: Data obtained with MDCK normal cells and used to calculate the selectivity index (SI) are reported in Table 1. **HL**: *N*-phenyl-2-[(pyridin-2-yl)methylidene]hydrazine-1-carbothioamide.

## Data Availability

Data are contained within the article and Supplementary Materials.

## Supplementary Materials

The following supporting information can be downloaded at: https://www.mdpi.com/article/10.3390/molecules29246002/s1, Figures S1–S10. ^1^H, ^13^C-NMR spectrum of thiosemicarbazone **HL^1−5^**. Figures S11–S15. FT-IR spectrum of **HL^1−5^**. Figures S16–S25. FT-IR spectrum of the coordination compounds [Cu(L^1^)Cl] (**C1**), [Cu(L^1^)NO_3_] (**C2**), [Cu(L^2^)Cl] (**C3**), [Cu(L^2^)NO_3_] (**C4**), [Cu(L^3^)Cl] (**C5**), [Cu(L^3^)NO_3_] (**C6**), [Cu(L^4^)NO_3_] (**C7**), [Cu(L^4^)Cl] (**C8**), [Cu(L^5^)Cl] (**C9**), and [Cu(L^5^)NO_3_] (**C10**). Table S1. Bond lengths (Å) and angles (deg) in **HL^2^**, **HL^3^**, **HL^5^**, and **C3a**. Table S2. Hydrogen bond distances (Å) and angles (deg) for **HL^2^**, **HL^3^**, **HL^5^**, and **C3a**.
