# Supplementary material for: Novel Copper(II) Coordination Compounds Containing Pyridine Derivatives of N4-Methoxyphenyl-Thiosemicarbazones with Selective Anticancer Activity
Source: Molecules. 2024 Dec 19;29(24):6002. doi: 10.3390/molecules29246002 (PMC11676775; doi:10.3390/molecules29246002)
Supplement: Supplementary file 1 [file molecules-29-06002-s001.zip › molecules-3360263-supplementary.pdf]

Supplementary materials

Novel Copper(II) Coordination Compounds Containing Pyridine Derivatives of N4-methoxyphenyl-Thiosemicarbazones with Selective Anticancer Activity

Roman Rusnac [1], Olga Garbuz [2,*], Victor Kravtsov [3], Elena Melnic [3], Dorin Istrati [4], Victor Tsapkov [1], Donald Poirier [5] and Aurelian Gulea [1,*]

1 Laboratory of Advanced Materials in Biopharmaceutics and Technics, Institute of Chemistry, Moldova State University, MD-2009 Chisinau, Moldova
2 Laboratory of Systematics and Molecular Phylogenetics, Institute of Zoology, Moldova State University, MD-2028 Chisinau, Moldova
3 Laboratory of Physical Methods of Solid State Investigation "Tadeusz Malinowski", Institute of Applied Physics, Moldova State University, MD-2028 Chisinau, Moldova
4 Department of Dentistry, University of Medicine and Pharmacy "Nicolae Testemitanu", MD-2004 Chisinau, Moldova
5 Laboratory of Medicinal Chemistry, CHU de Québec Research Center, Université Laval, Québec, QC G1V 4G2, Canada; donald.poirier@crchudequebec.ulaval.ca
* Correspondence: olhamos1@gmail.com or olga.garbuz@sti.usm.md (O.G.); aurelian.gulea@usm.md or guleaaurelian@gmail.com (A.G.)

Content

Figure S1. ^{1}H-NMR spectrum of thiosemicarbazone HL^1. 3

Figure S2. ^{13}C-NMR spectrum of thiosemicarbazone HL^1. 4

Figure S3. ^{1}H-NMR spectrum of thiosemicarbazone HL^2. 5

Figure S4. ^{13}C-NMR spectrum of thiosemicarbazone HL^2. 6

Figure S5. ^{1}H-NMR spectrum of thiosemicarbazone HL^3. 7

Figure S6. ^{13}C-NMR spectrum of thiosemicarbazone HL^3. 8

Figure S7. ^{1}H-NMR spectrum of thiosemicarbazone HL^4. 9

Figure S8. ^{13}C-NMR spectrum of thiosemicarbazone HL^4. 10

Figure S9. ^{1}H-NMR spectrum of thiosemicarbazone HL^5. 11

Figure S10. ^{13}C-NMR spectrum of thiosemicarbazone HL^5. 12

Figure S11. FT-IR spectrum of HL^1.......... 13

Figure S12. FT-IR spectrum of HL^2.......... 14

Figure S13. FT-IR spectrum of HL^3.......... 15

Figure S14. FT-IR spectrum of HL^4.......... 16

Figure S15. FT-IR spectrum of HL^5.......... 17

Figure S16. FT-IR spectrum of the coordination compound $[Cu(L^1)Cl]$ (C1) 18

Figure S17. FT-IR spectrum of the coordination compound $[Cu(L^1)NO_3]$ (C2).......... 19

Figure S18. FT-IR spectrum of the coordination compound $[Cu(L^2)Cl]$ (C3) 20

Figure S19. FT-IR spectrum of the coordination compound $[Cu(L^2)NO_3]$ (C4).......... 21

Figure S20. FT-IR spectrum of the coordination compound [Cu(L^3)Cl] (C5) 22
Figure S21. FT-IR spectrum of the coordination compound [Cu(L^3)NO_3] (C6) 23
Figure S22. FT-IR spectrum of the coordination compound [Cu(L^4)NO_3] (C7) 24
Figure S23. FT-IR spectrum of the coordination compound [Cu(L^4)Cl] (C8) 25
Figure S24. FT-IR spectrum of the coordination compound [Cu(L^5)Cl] (C9) 26
Figure S25. FT-IR spectrum of the coordination compound [Cu(L^5)NO_3] (C10) 27

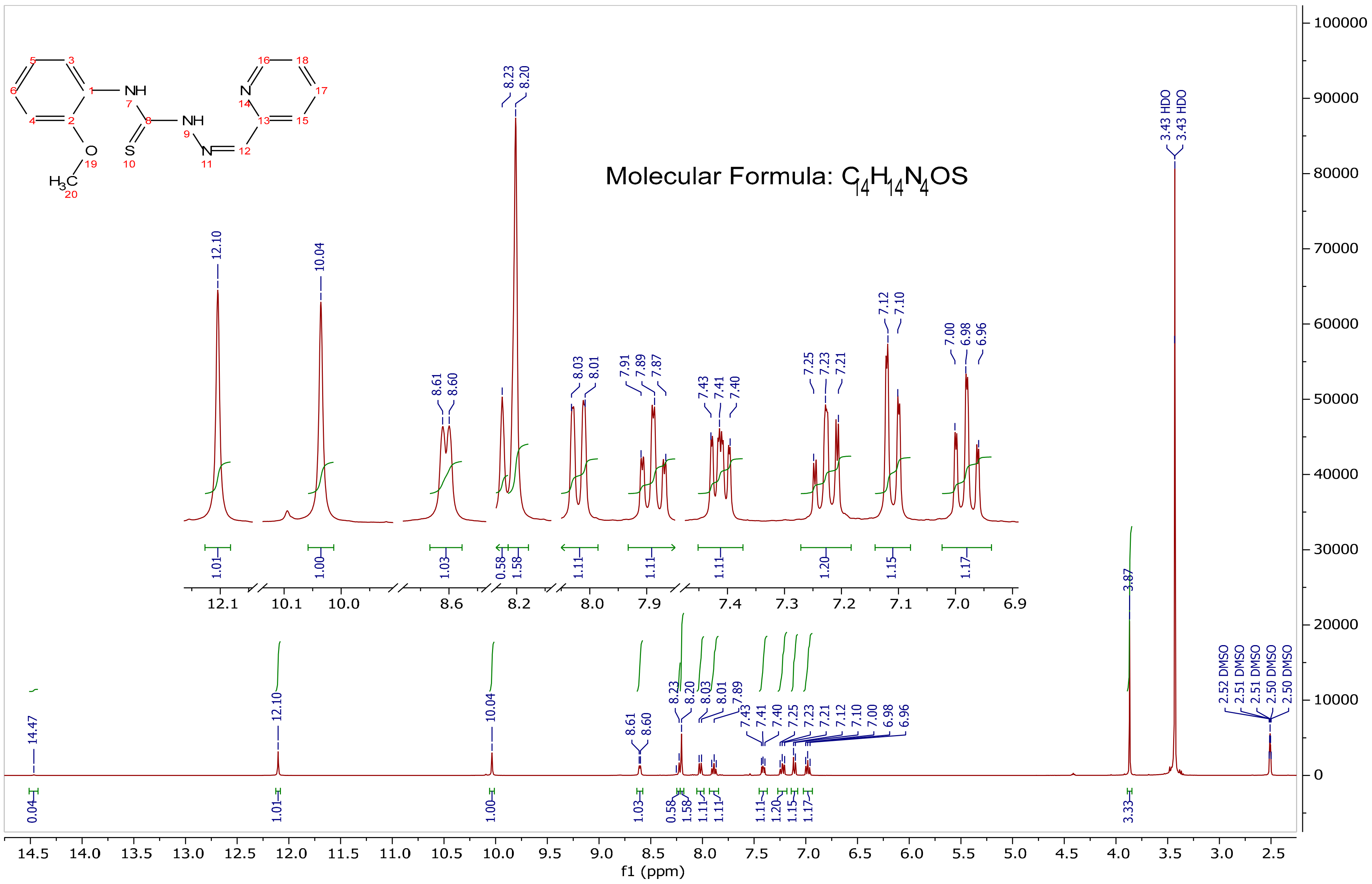

Figure S1. ^{1}H-NMR spectrum of thiosemicarbazone HL[1].

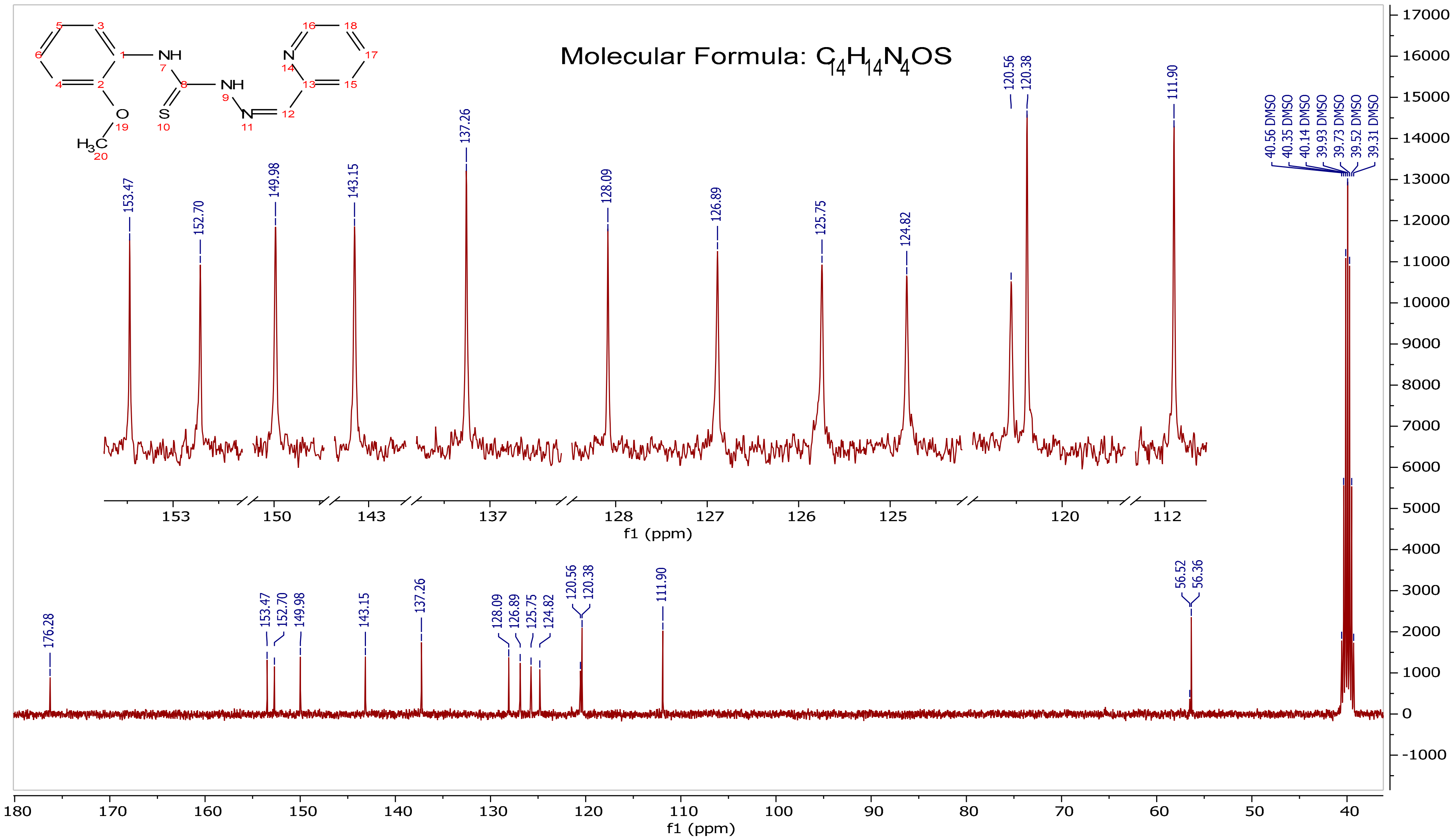

Figure S2. ^{13}**C-NMR spectrum of thiosemicarbazone HL**^{1}**.**

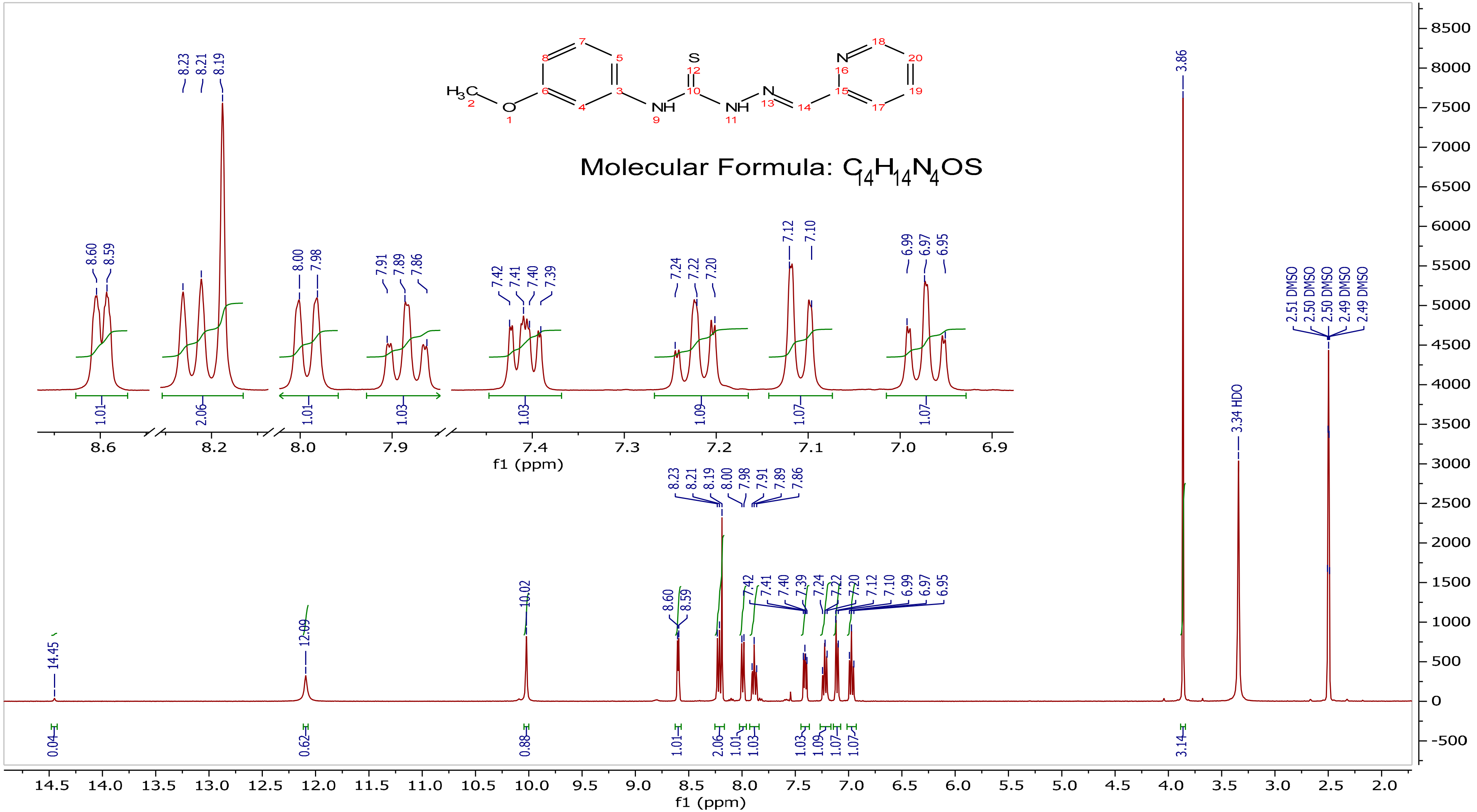

Figure S3. ^{1}H-NMR spectrum of thiosemicarbazone HL^{2}.

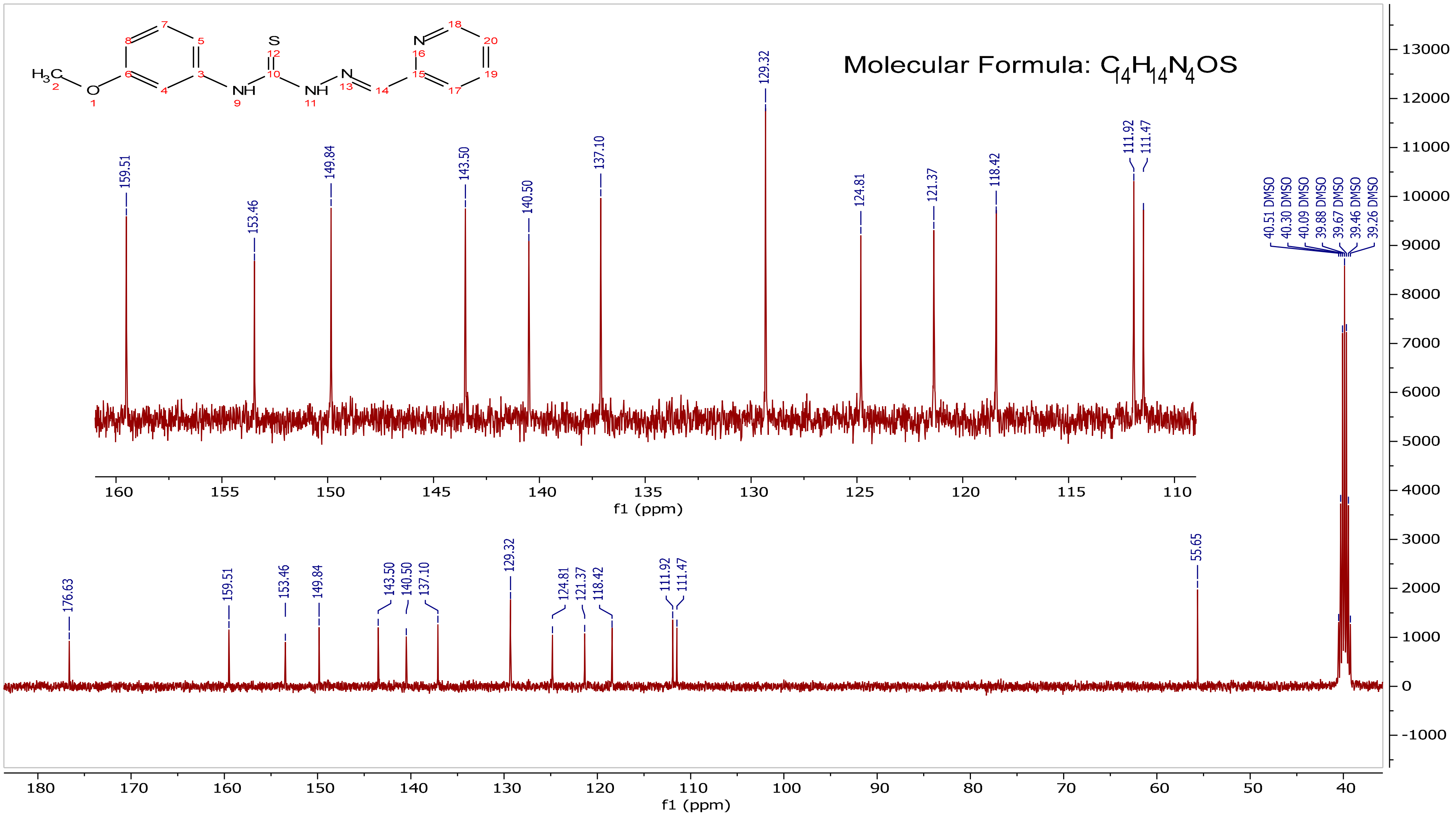

Figure S4. ^{13}**C-NMR spectrum of thiosemicarbazone HL**^{2}**.**

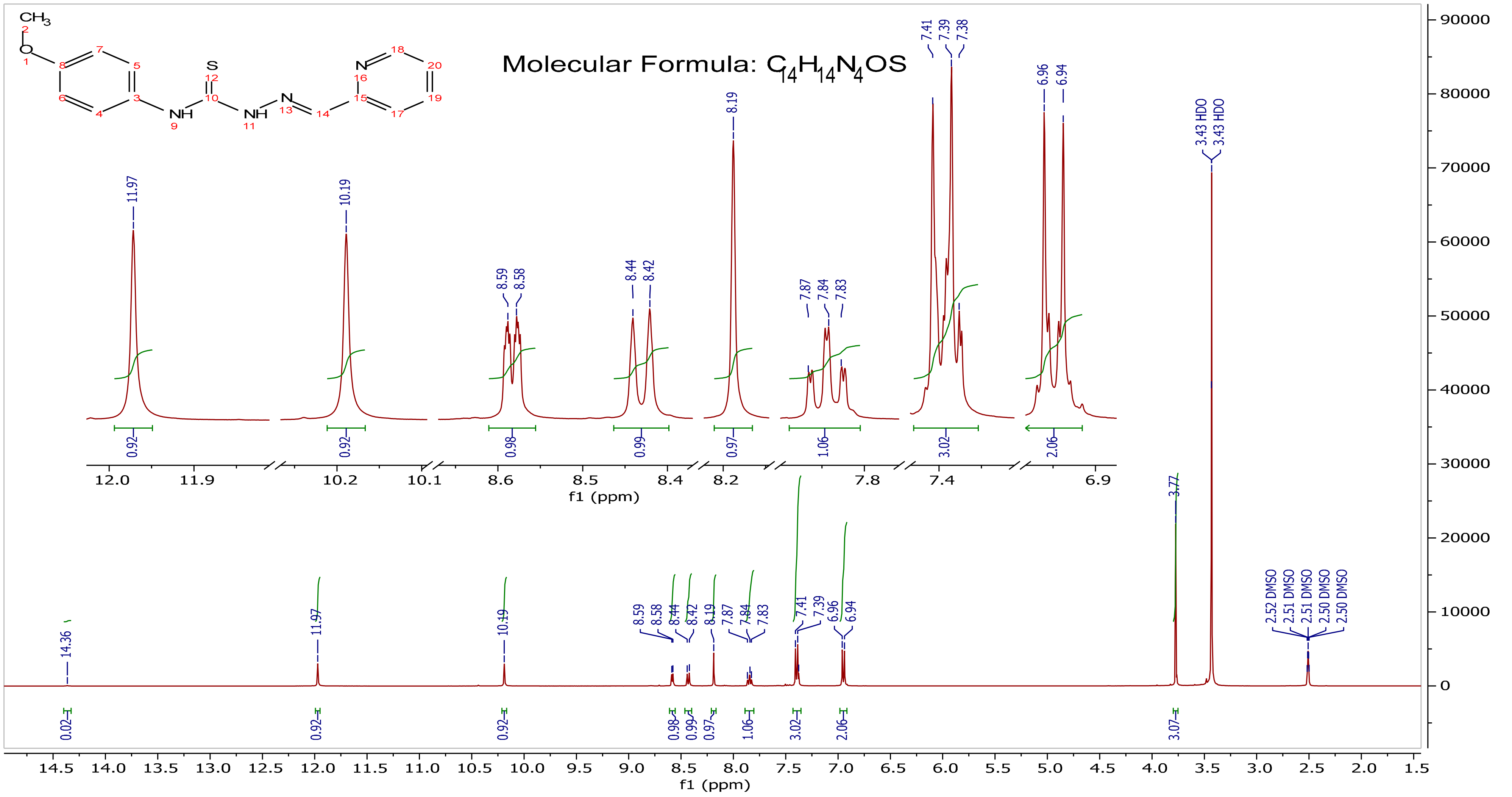

Figure S5. ^{1}H-NMR spectrum of thiosemicarbazone HL^3.

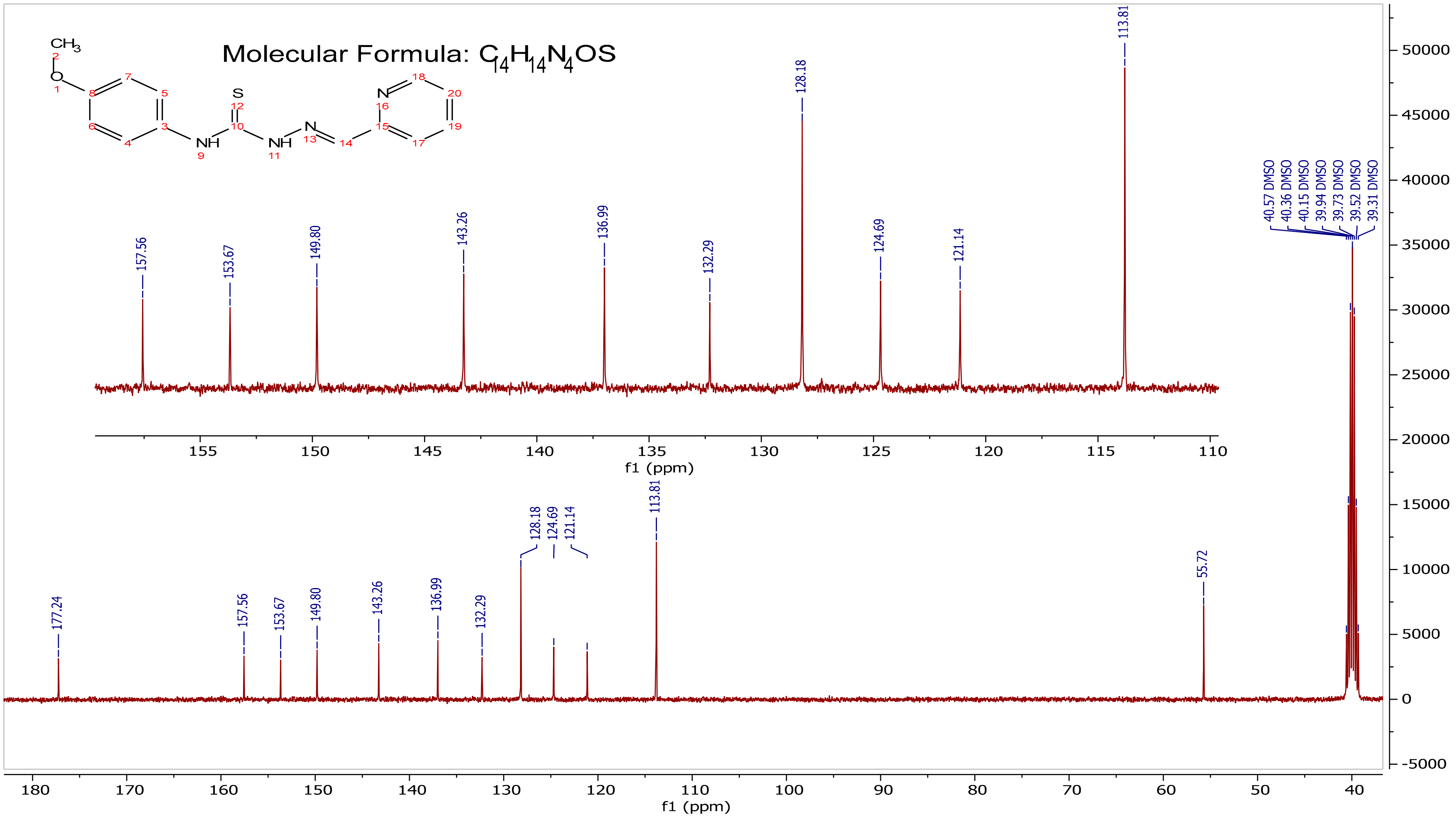

Figure S6. ^{13}**C-NMR spectrum of thiosemicarbazone HL**^{3}**.**

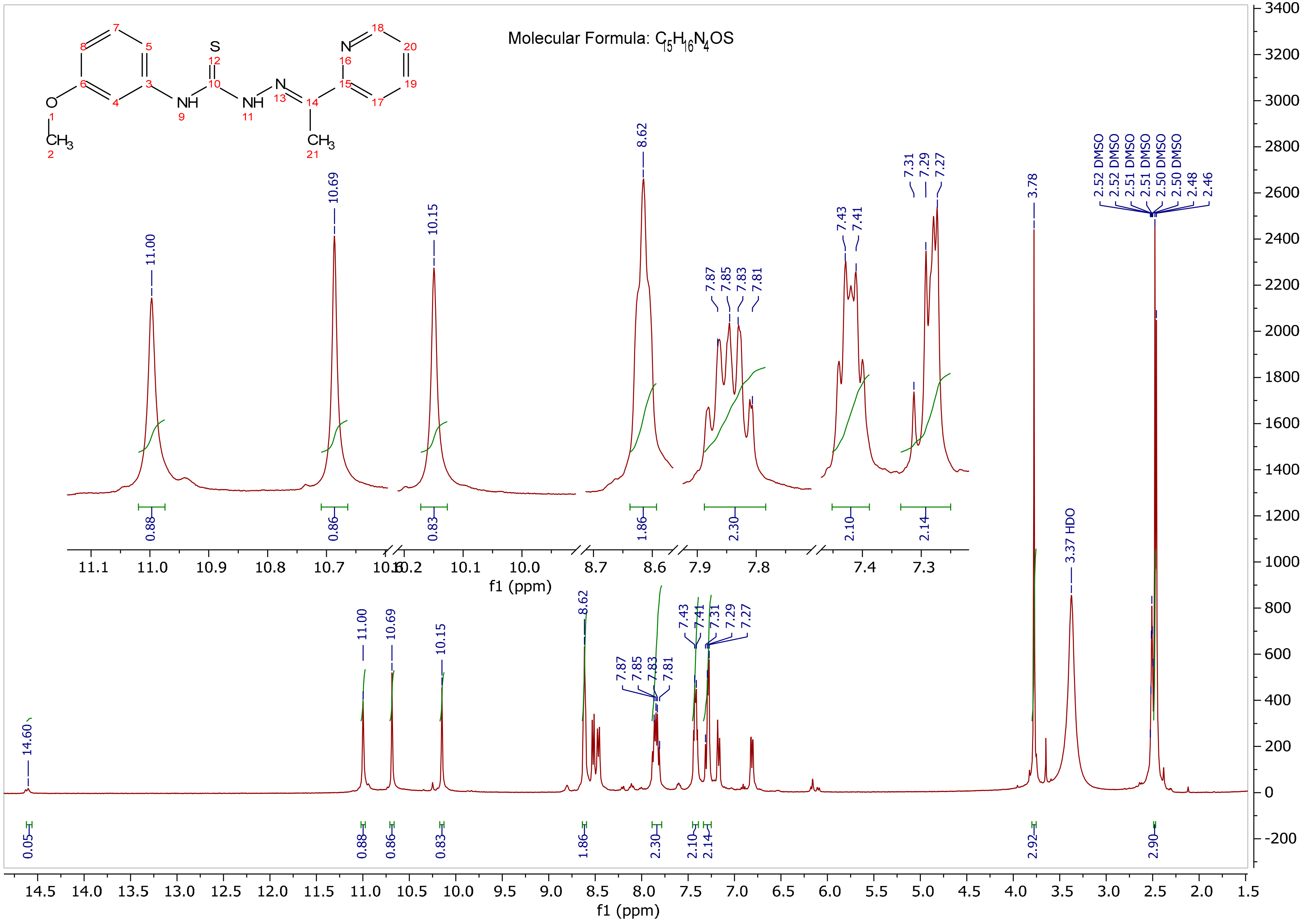

Figure S7. ^{1}H-NMR spectrum of thiosemicarbazone HL^4.

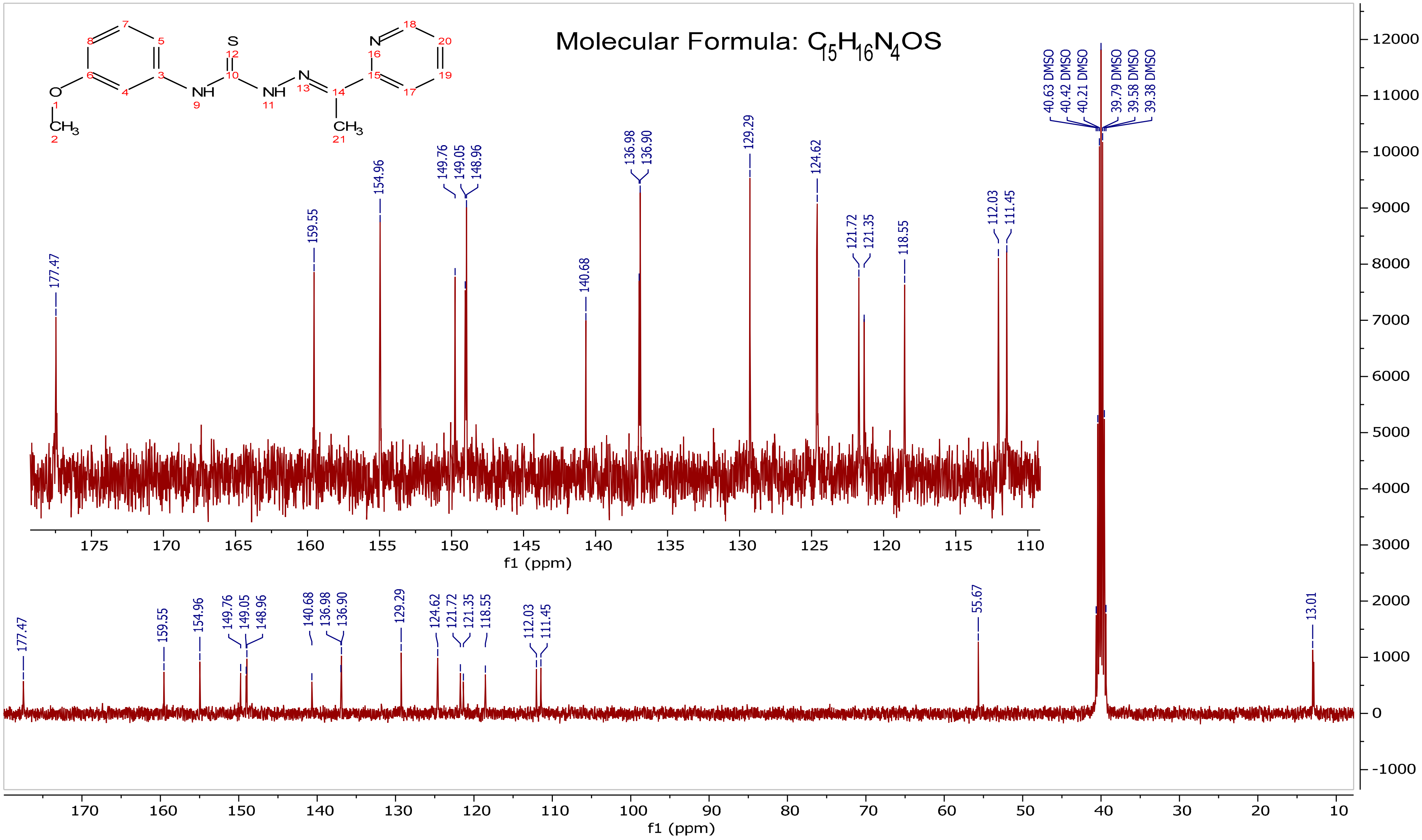

Figure S8. ^{13}C-NMR spectrum of thiosemicarbazone HL^{4}.

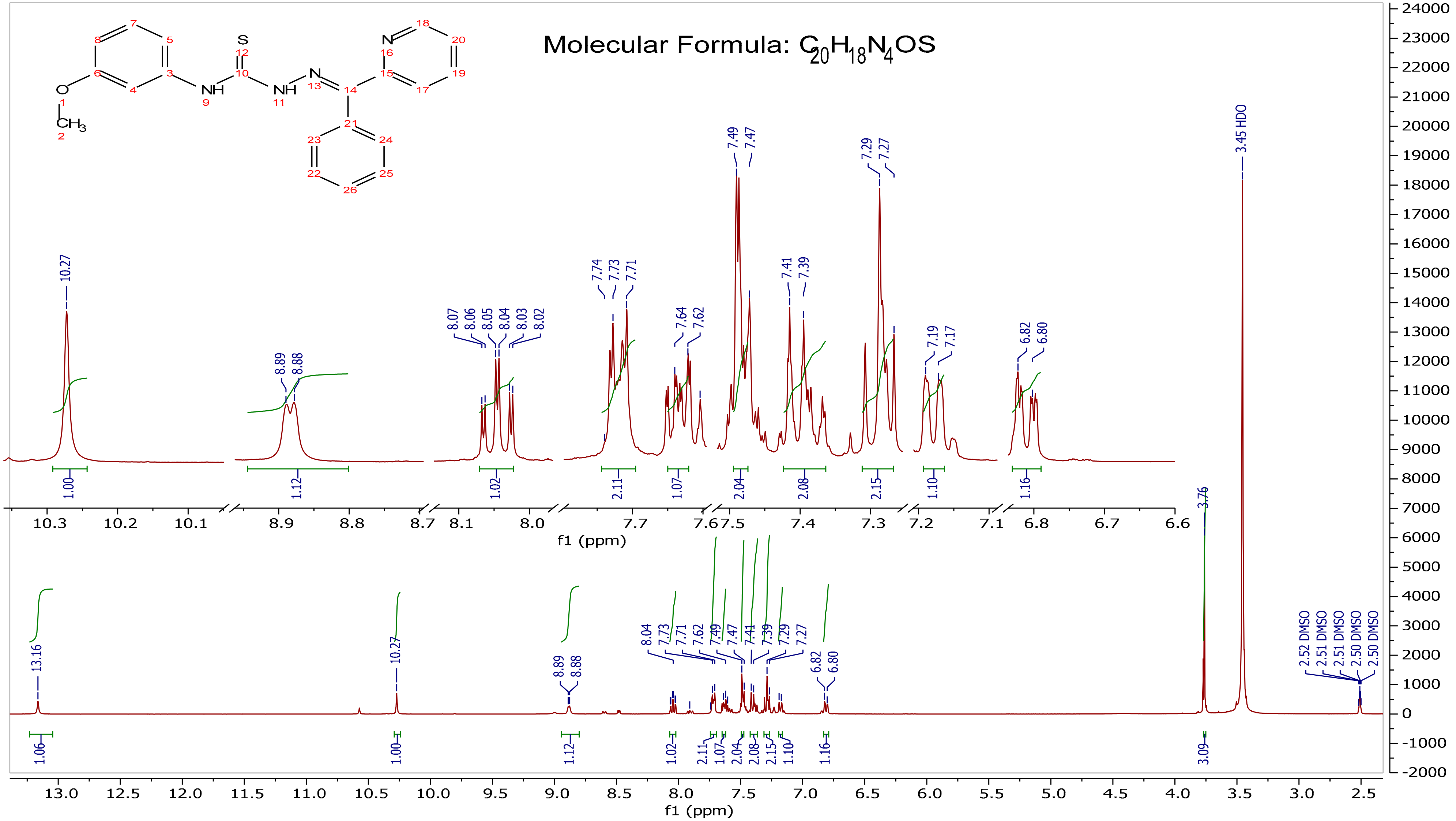

Figure S9. ^{1}H-NMR spectrum of thiosemicarbazone HL[5].

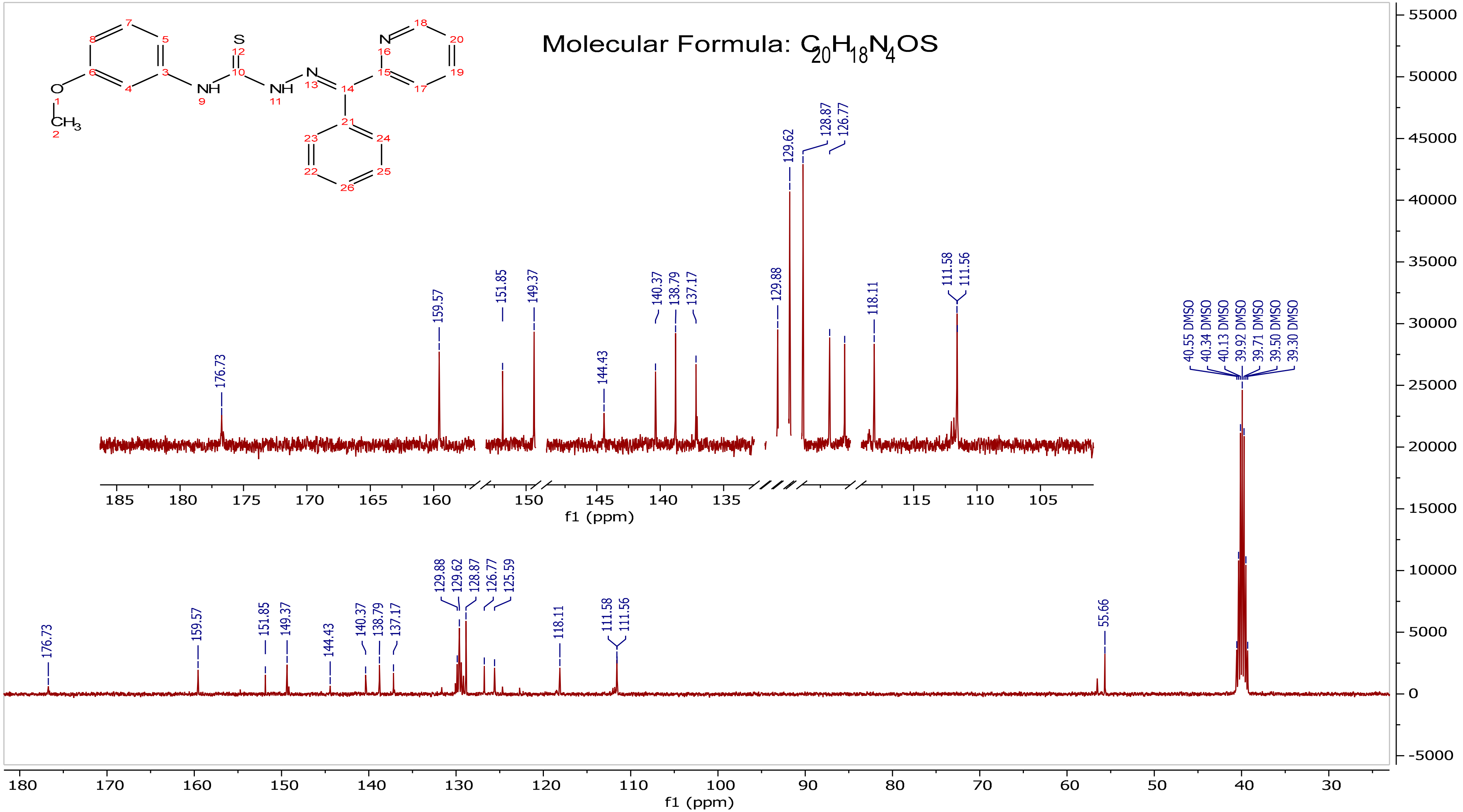

Figure S10. ^{13}C-NMR spectrum of thiosemicarbazone HL^{5}.

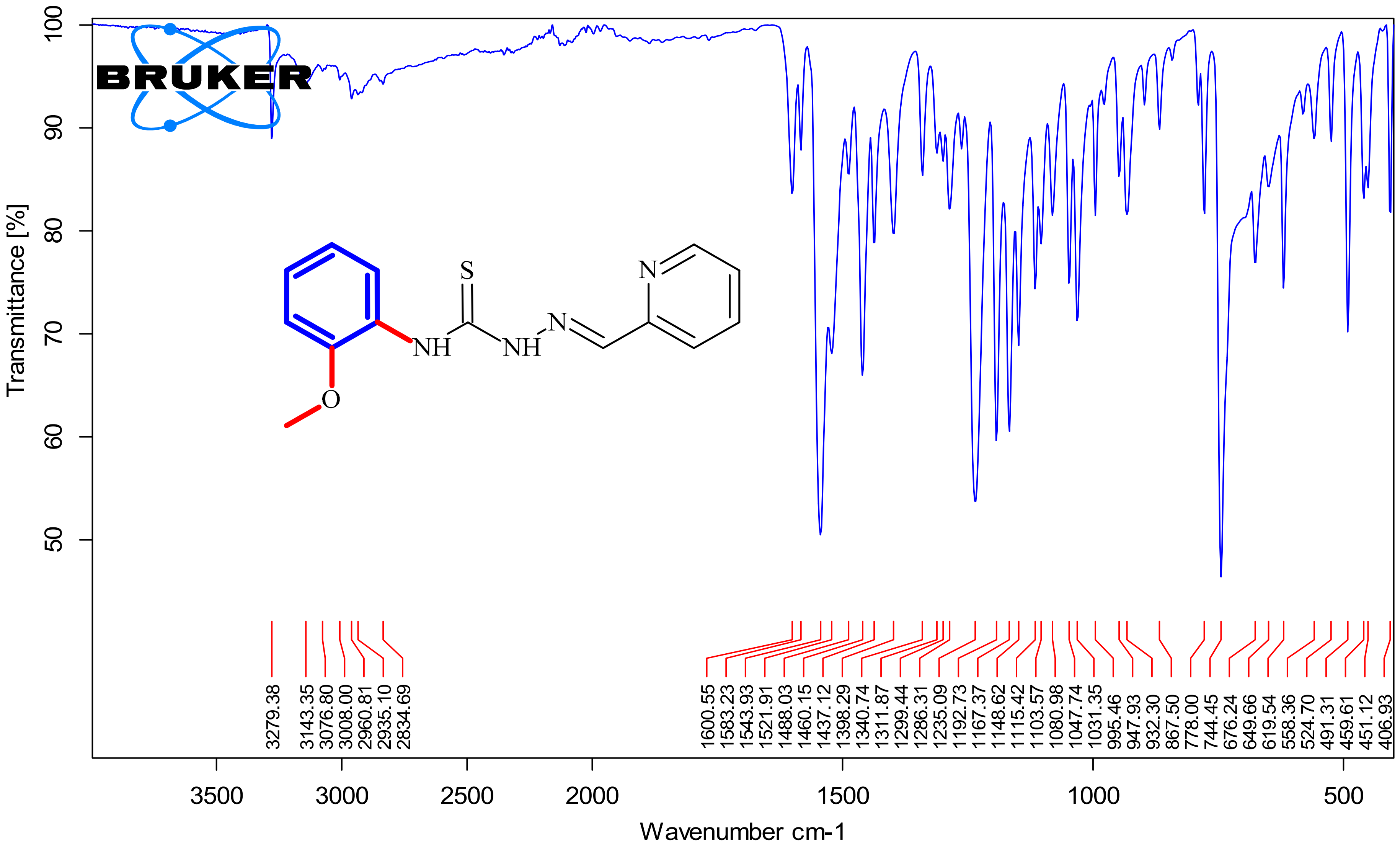

Figure S11. FT-IR spectrum of HL[1].

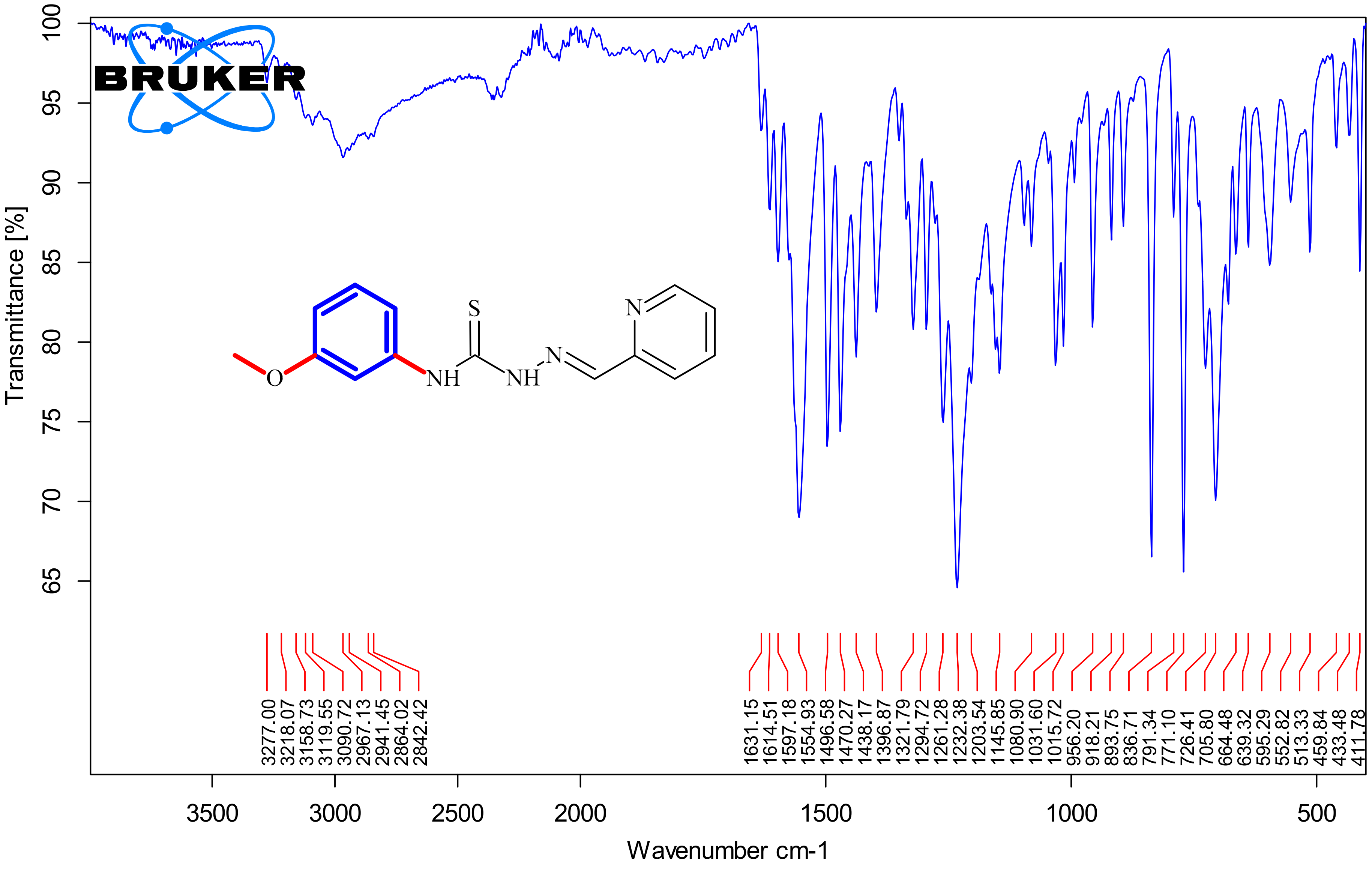

Figure S12. FT-IR spectrum of HL^2.

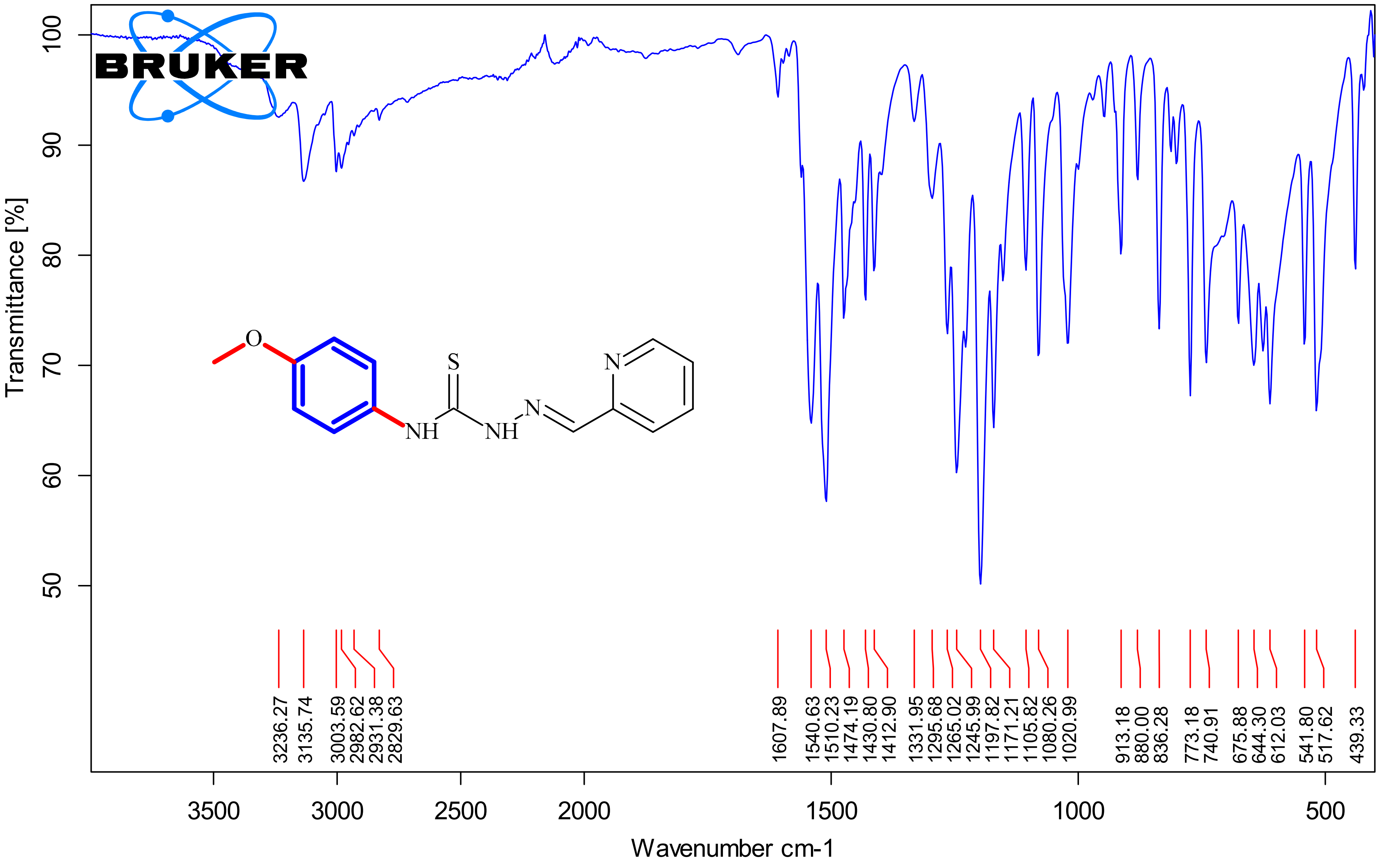

Figure S13. FT-IR spectrum of HL^3.

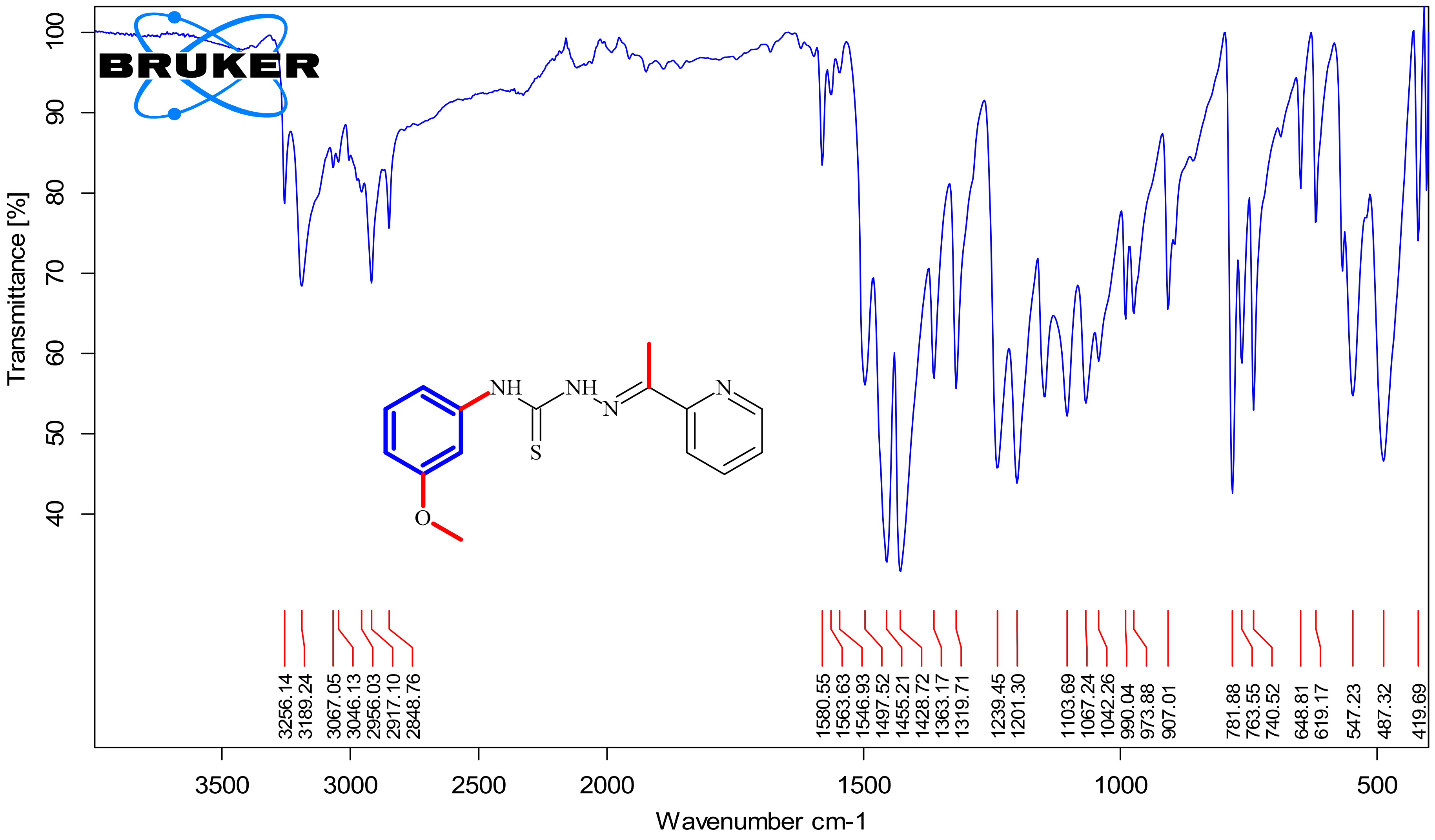

Figure S14. FT-IR spectrum of HL[4].

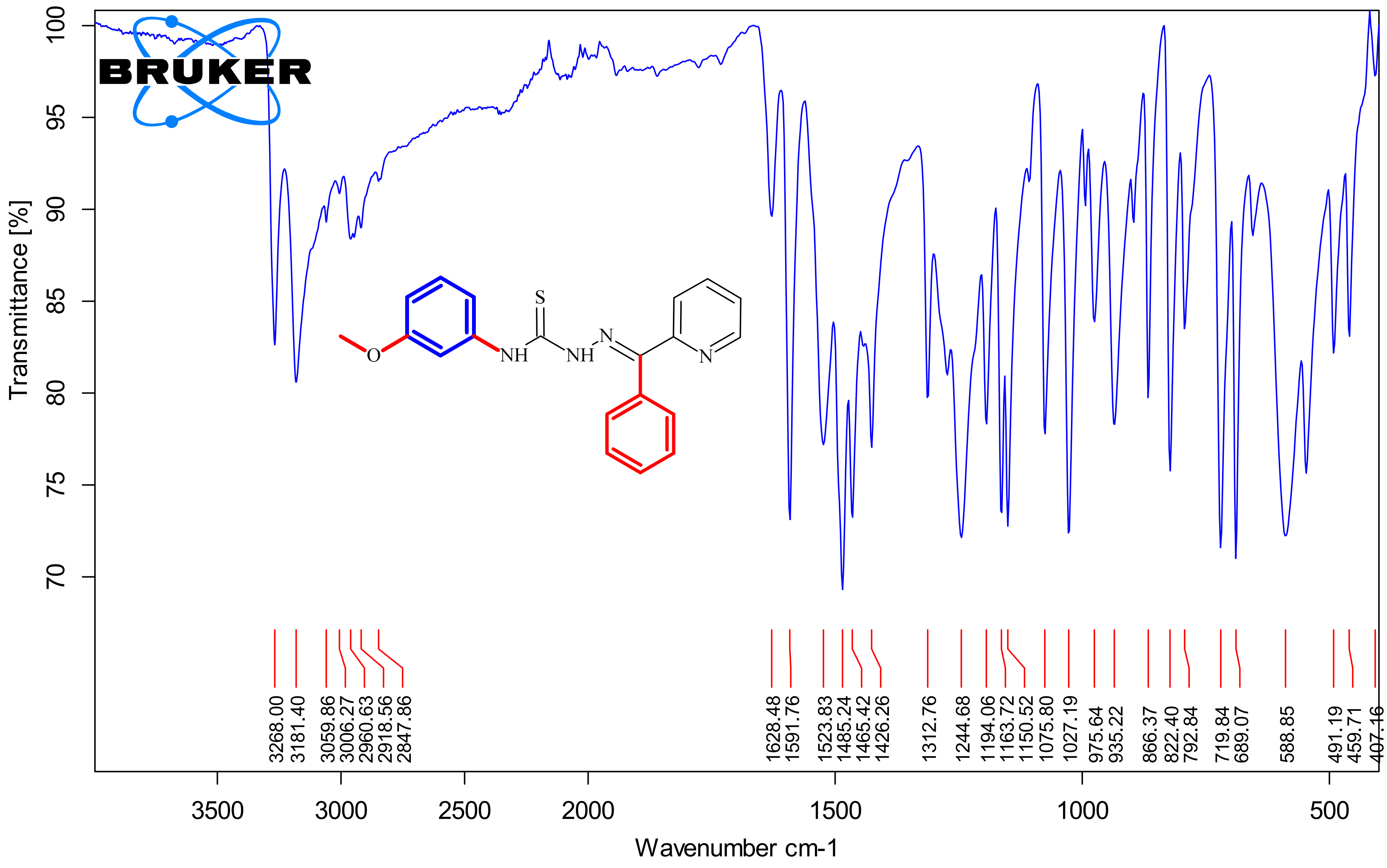

Figure S15. FT-IR spectrum of HL[5].

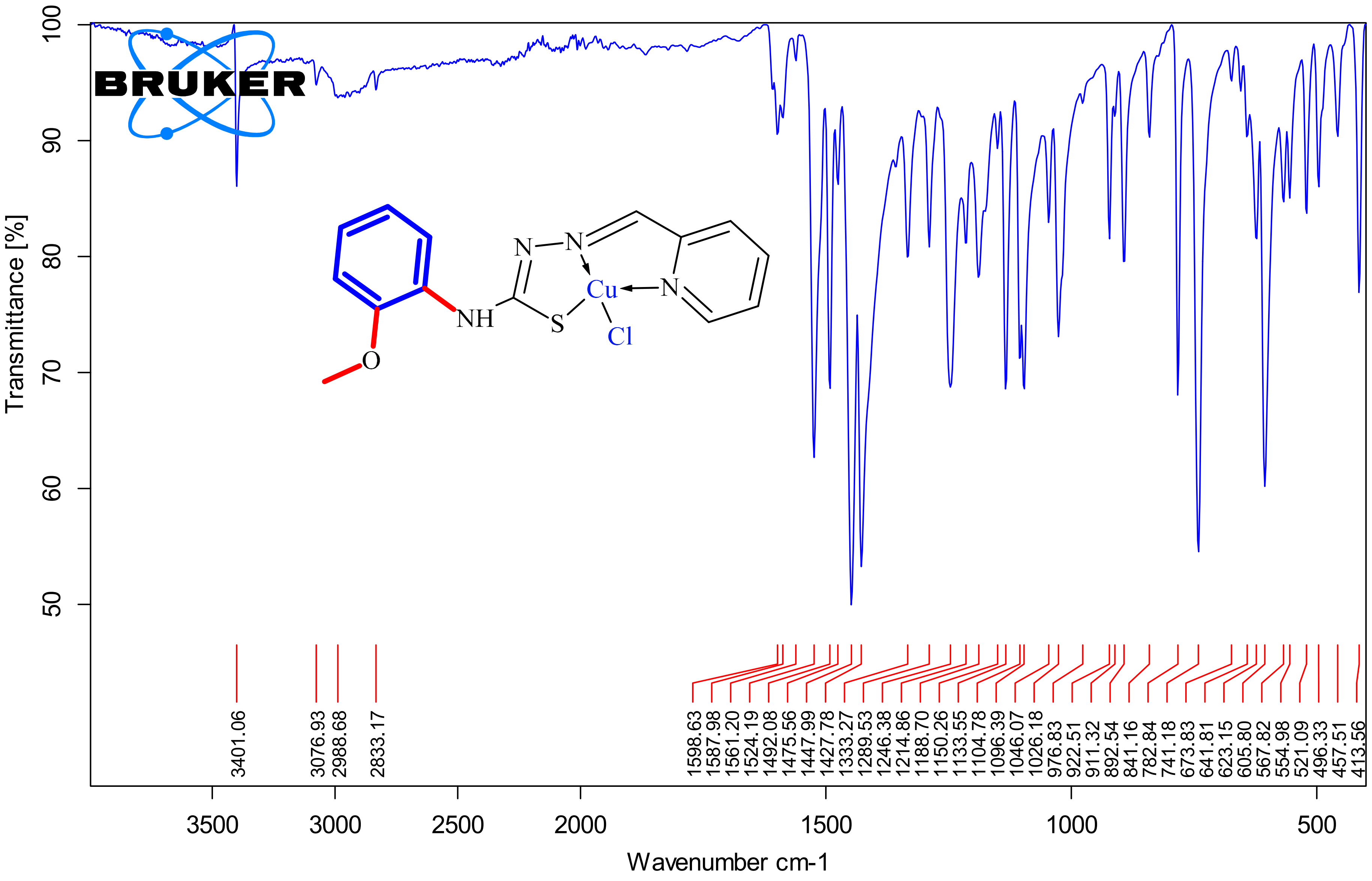

Figure S16. FT-IR spectrum of the coordination compound $[Cu(L^1)Cl]$ **(C1)**

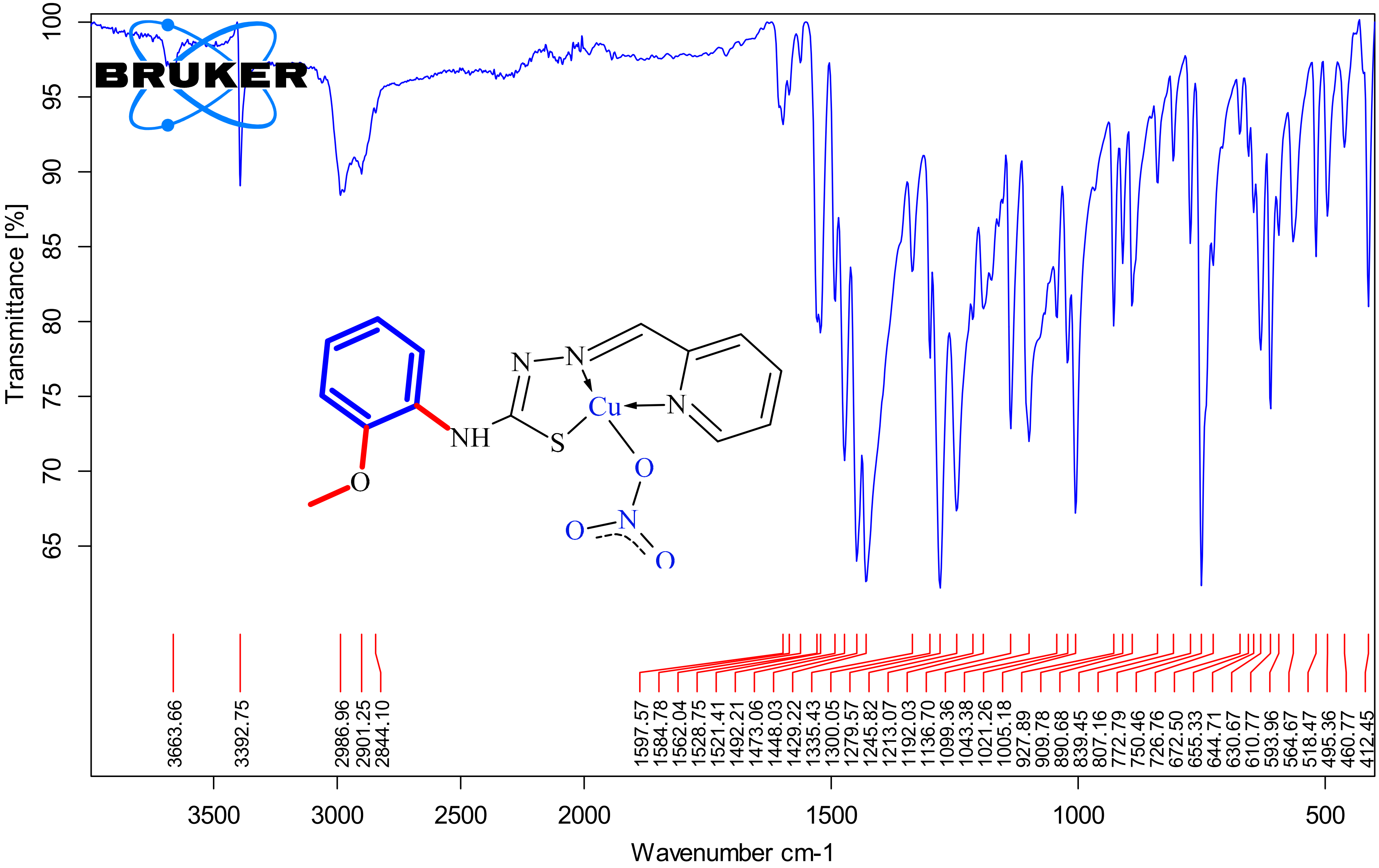

Figure S17. FT-IR spectrum of the coordination compound [Cu(L[1])NO3] (C2)

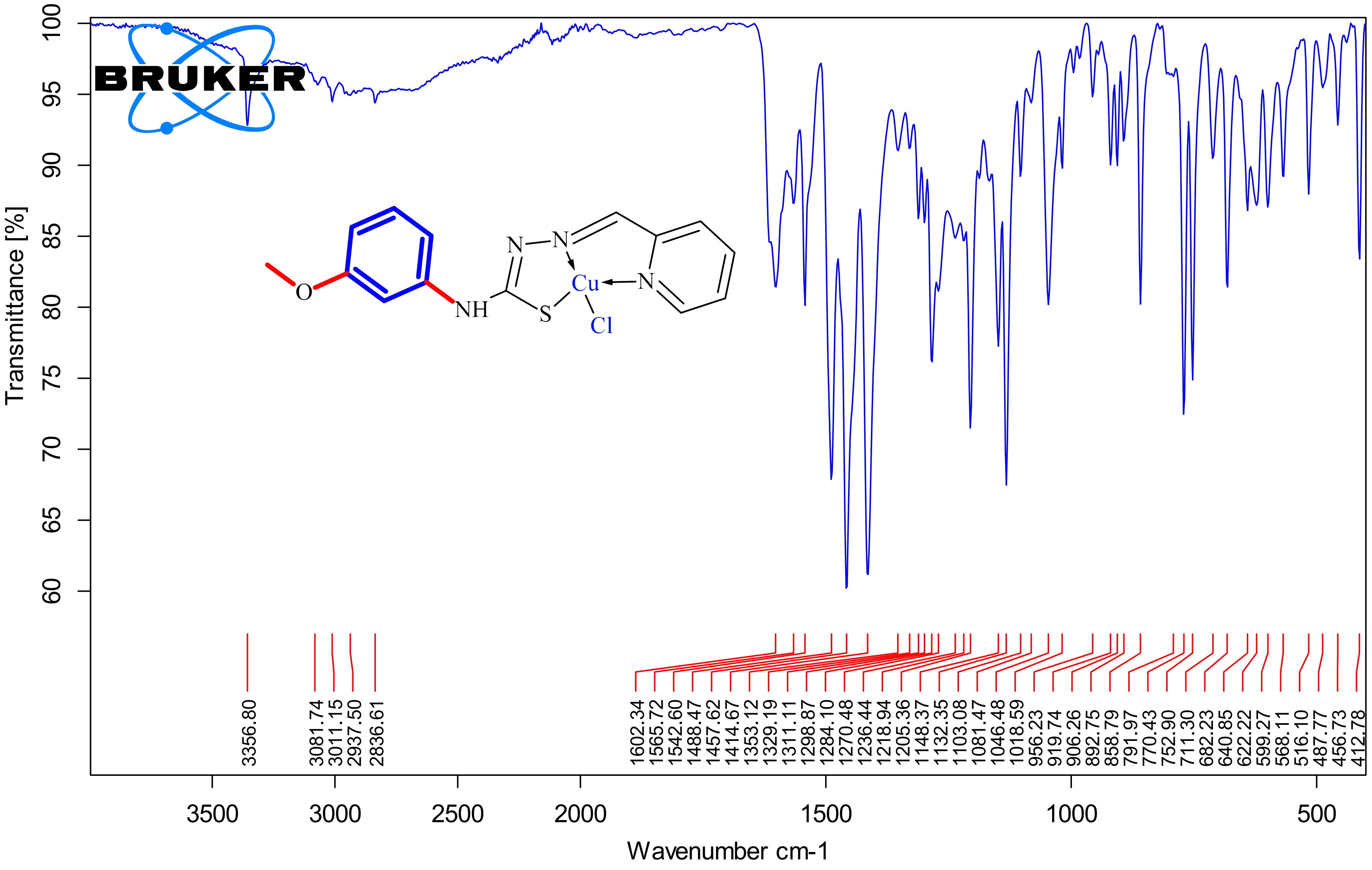

Figure S18. FT-IR spectrum of the coordination compound [Cu(L^2)Cl] (C3)

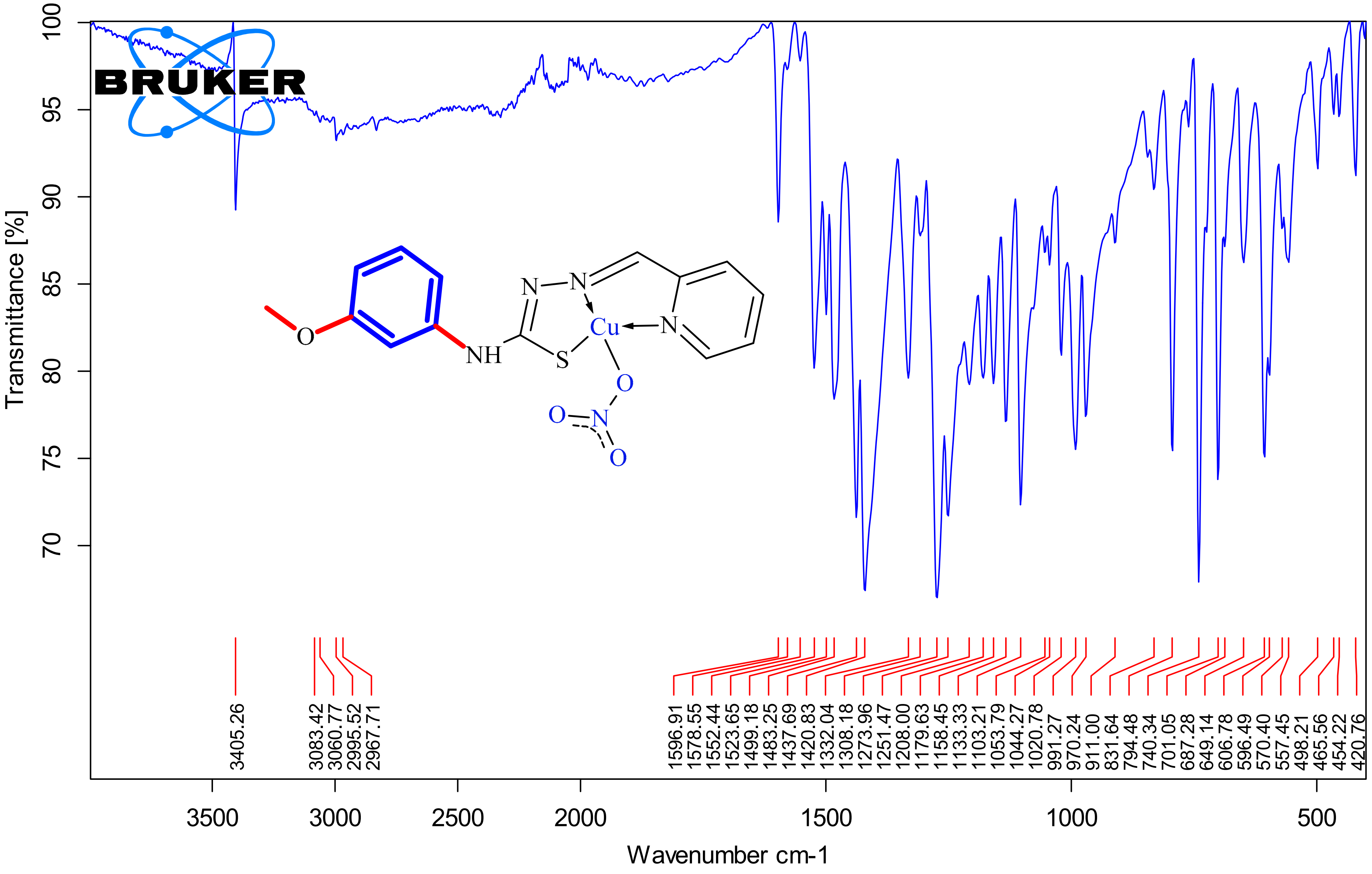

Figure S19. FT-IR spectrum of the coordination compound [Cu(L²)NO₃] (C4)

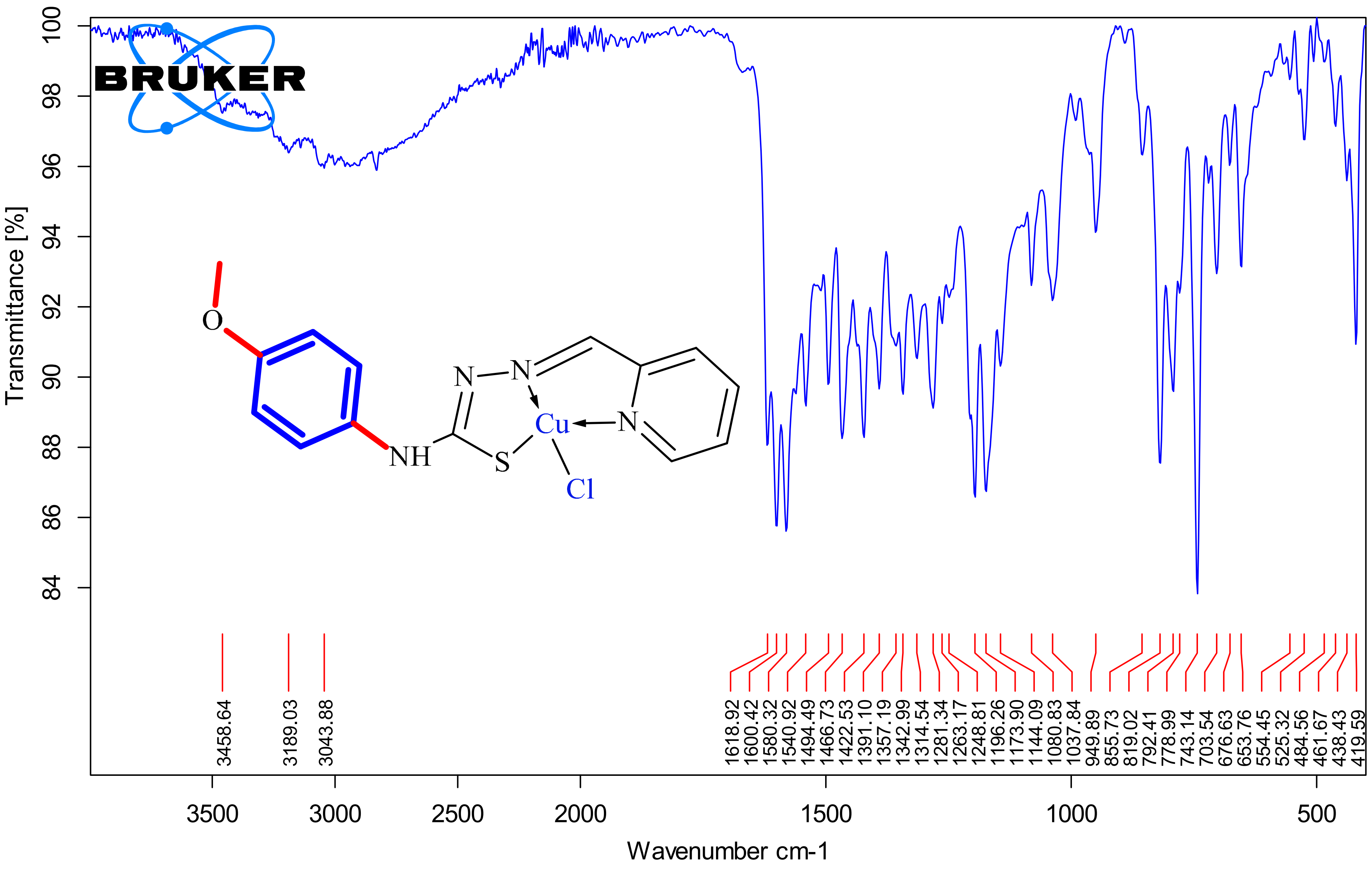

Figure S20. FT-IR spectrum of the coordination compound [Cu(L³)Cl] (C5)

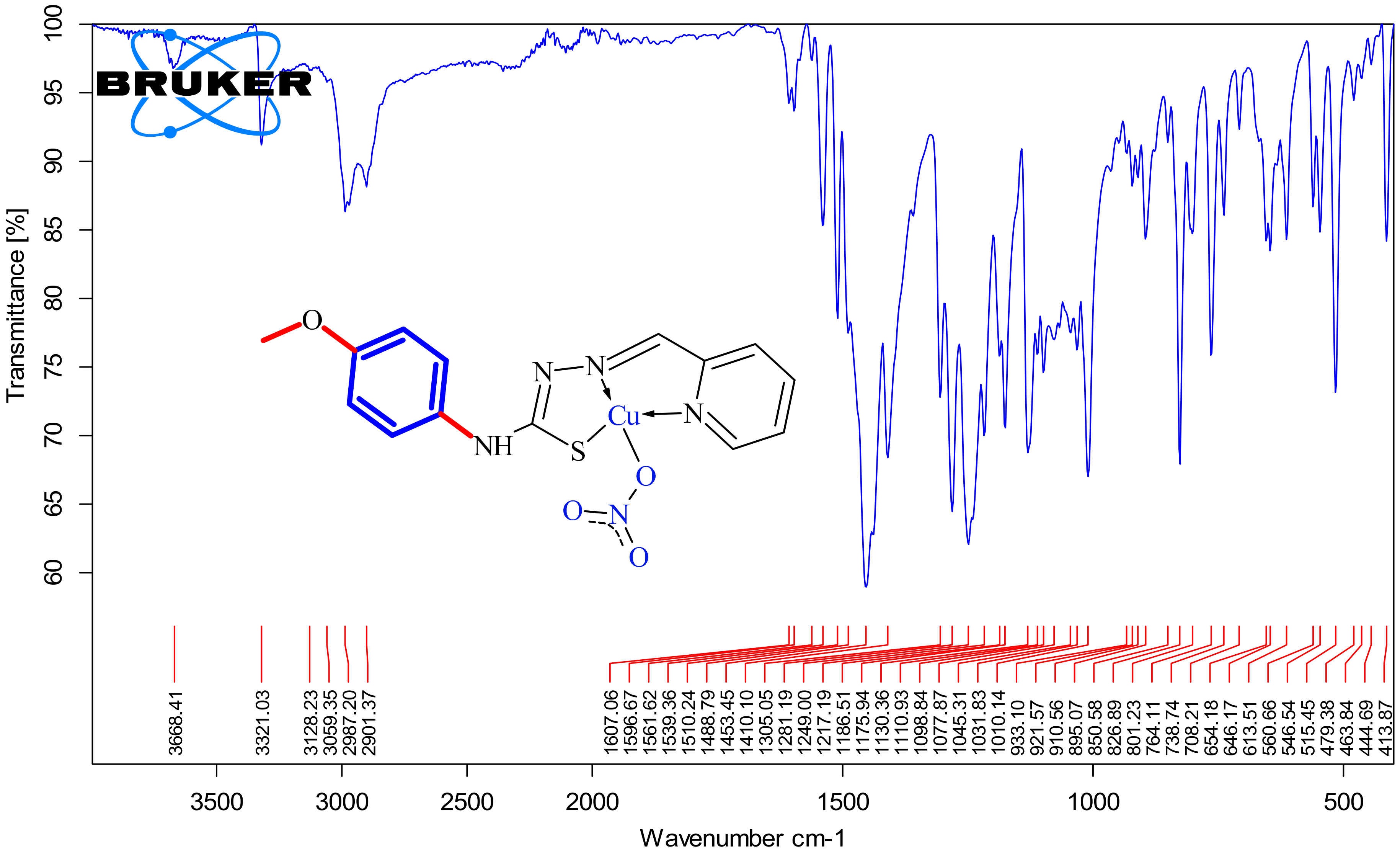

Figure S21. FT-IR spectrum of the coordination compound [Cu(L³)NO₃] (C6)

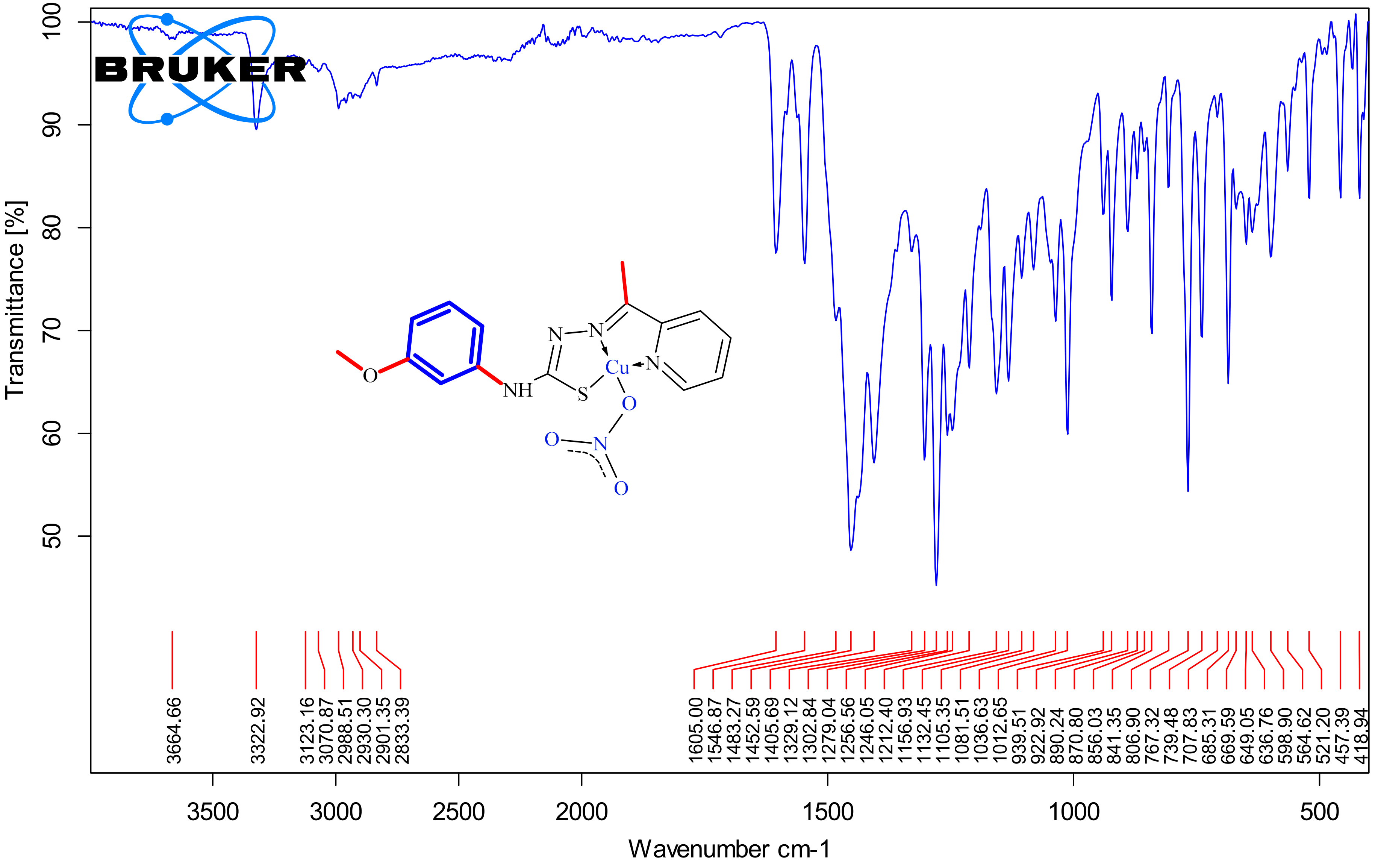

Figure S22. FT-IR spectrum of the coordination compound [Cu(L4)NO3] (C7)

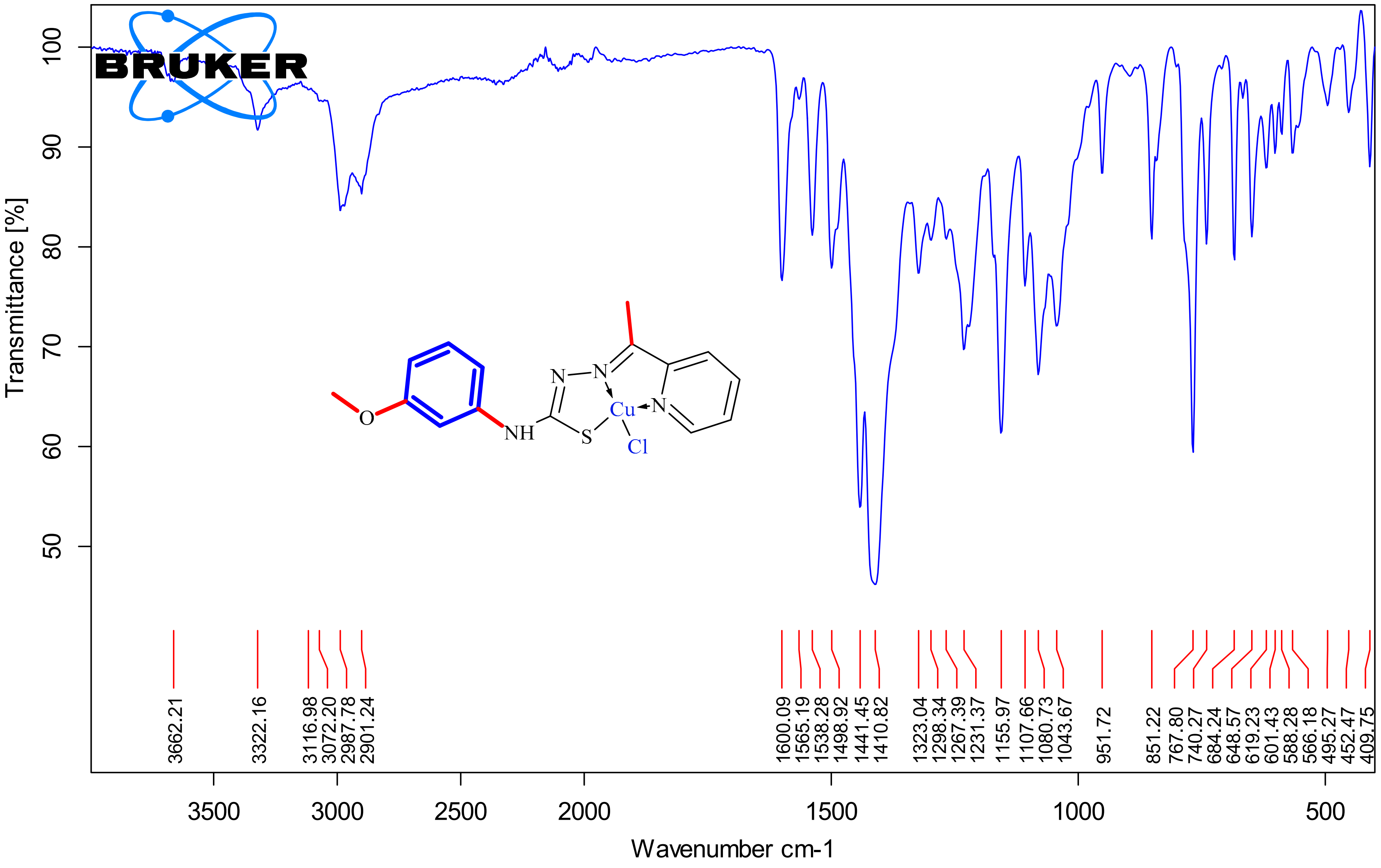

Figure S23. FT-IR spectrum of the coordination compound **[Cu(L⁴)Cl] (C8)**

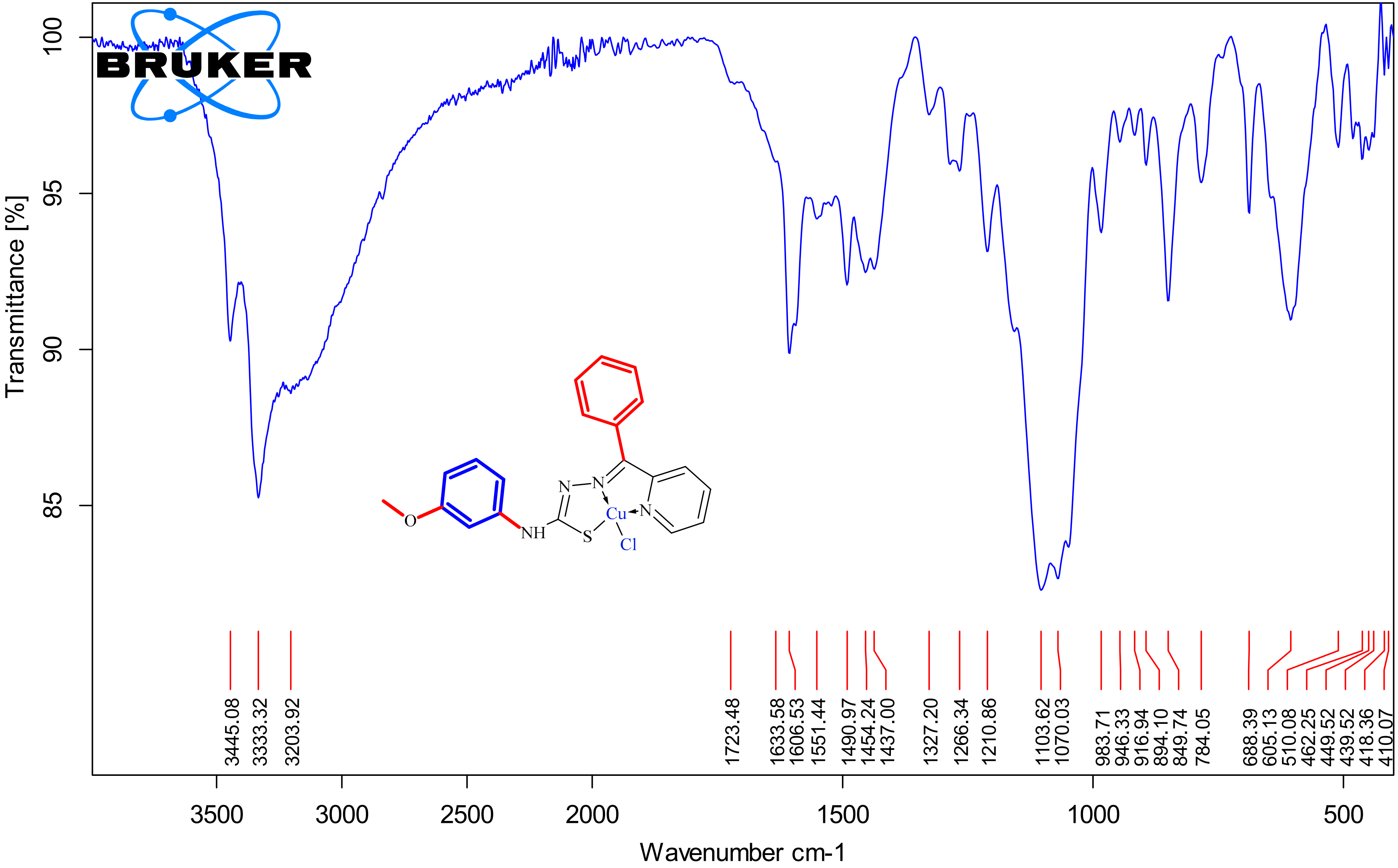

Figure S24. FT-IR spectrum of the coordination compound [Cu(L⁵)Cl] (C9)

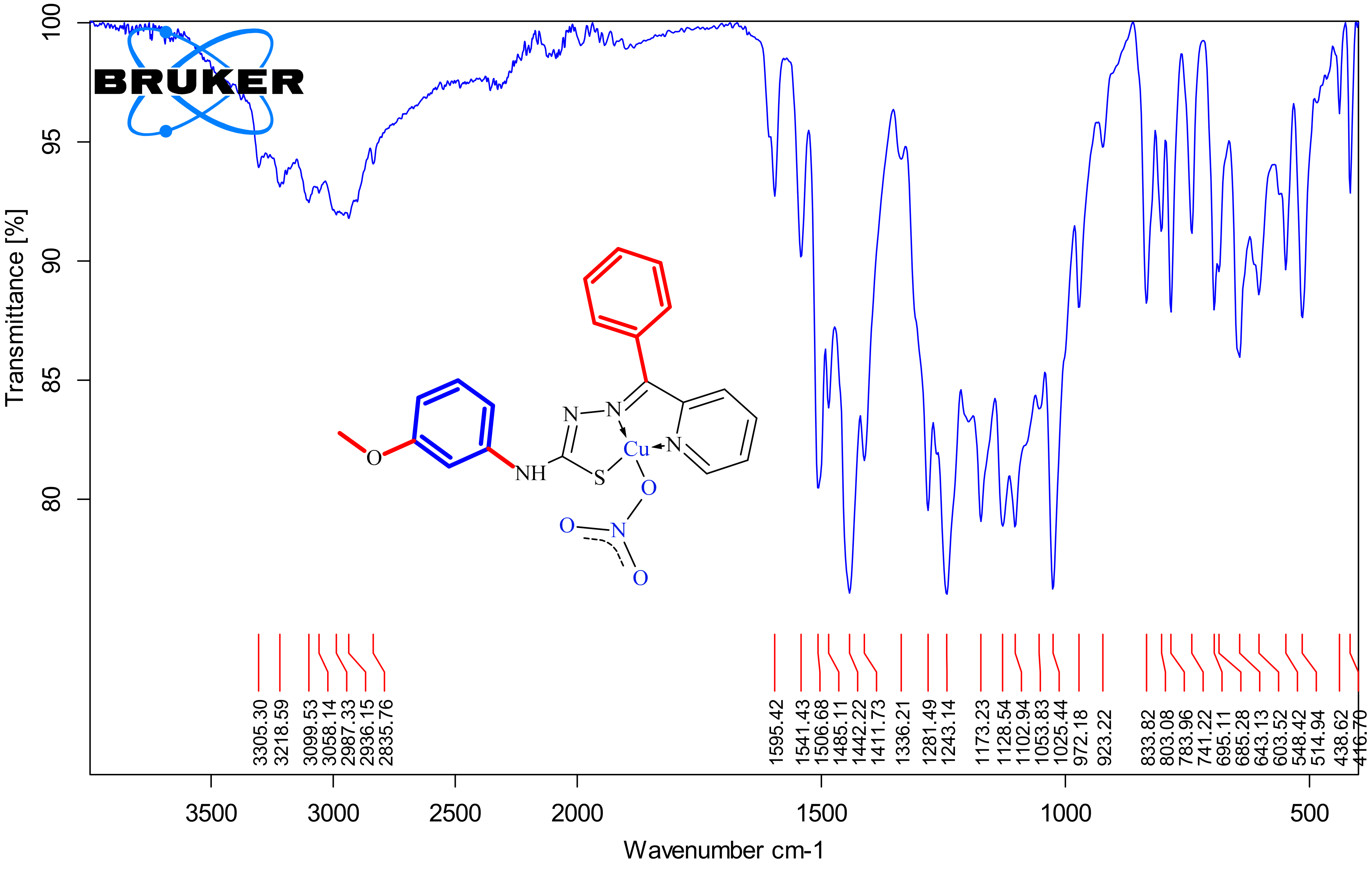

Figure S25. FT-IR spectrum of the coordination compound **[Cu(L[5])NO3] (C10)**

Table S1. Bond Lengths (Å) and Angles (deg) in **HL²**,**HL³**, **HL⁵** and **C3a**

**Bonds**	**HL²**, (Å)	**HL³**, (Å)		**HL⁵**, (Å)	**C3a**, (Å)
		*molecule **A***	*molecule **B***		
C(1)-N(1)	1.350(3)	1.317(7)	1.322(6)	1.342(3)	1.363(5)
C(1)-N(2)	1.357(3)	1.357(7)	1.362(7)	1.366(3)	1.312(5)
C(1)-S(1)	1.658(3)	1.679(7)	1.690(6)	1.661(2)	1.747(5)
C(2)-N(1)	1.420(3)	1.417(7)	1.413(7)	1.409(3)	1.401(6)
C(6)-O(1)	1.367(4)	1.386(7)	1.352(7)		
C(4)-O(1)				1.371(3)	1.359(5)
C(8)-O(1)	1.428(4)	1.421(7)	1.429(7)	1.428(3)	1.406(7)
C(10)-N(4)	1.339(3)	1.338(7)	1.342(7)	1.348(3)	1.345(6)
C(11)-N(4)				1.331(3)	1.331(6)
N(2)-N(3)	1.375(3)	1.382(7)	1.342(7)	1.359(2)	1.365(5)
Cu(1)-N(3)					1.962(4)
Cu(1)-N(4)					2.045(3)
Cu(1)-Cl(1)					2.2362(12)
Cu(1)-S(1)					2.2565(13)
**Angles**	**HL²**, (°)	**HL³**, (°)		**HL⁵**, (°)	**3a**, (°)
		*molecule **A***	*molecule **B***		
N(1)-C(1)-N(2)	113.8(3)	116.7(6)	115.8(6)	113.2(2)	119.5(4)
N(1)-C(1)-S(1)	128.0(2)	126.6(5)	126.7(5)	129.2(2)	115.5(3)
N(2)-C(1)-S(1)	118.1(2)	116.6(5)	117.4(5)	117.6(2)	125.1(3)
C(7)-C(2)-N(1)	116.6(3)	119.4(6)	118.9(6)	115.6(2)	124.7(4)
C(3)-C(2)-N(1)	123.0(3)	121.3(6)	123.3(6)	124.9(2)	115.4(4)
C(5)-C(6)-O(1)	124.5(3)				
C(6)-C(5)-O(1)		116.5(6)	117.2(6)		
O(1)-C(6)-C(7)	115.4(4)				
O(1)-C(4)-C(3)				114.4(2)	125.2(5)
C(4)-C(5)-O(1)		123.2(6)	123.9(6)	124.0(2)	

N(3)-C(9)-C(10)	121.4(3)	120.5(7)	120.7(6)	126.6(2)	117.1(4)
N(4)-C(10)-C(14)	123.1(3)	123.5(7)	121.6(7)	121.0(2)	121.8(4)
N(4)-C(10)-C(9)	114.4(3)	117.5(7)	117.9(6)	117.1(2)	114.9(4)
N(4)-C(11)-C(12)	123.5(3)	123.4(7)	124.2(7)	123.8(3)	121.4(5)
C(1)-N(1)-C(2)	133.3(3)	126.3(6)	126.2(6)	133.6(2)	131.3(4)
C(1)-N(2)-N(3)	121.8(2)	119.5(6)	120.0(5)	119.9(2)	111.2(4)
C(9)-N(3)-N(2)	115.8(2)	115.6(6)	117.2(6)	120.2(2)	119.6(4)
C(11)-N(4)-C(10)	117.0(3)	116.6(7)	117.8(6)	118.3(2)	119.1(4)
C(6)-O(1)-C(8)	117.4(3)				
C(5)-O(1)-C(8)		116.2(5)	118.8(5)		
C(4)-O(1)-C(8)				117.9(2)	118.3(4)
C(9)-N(3)-Cu(1)					115.6(3)
C(11)-N(4)-Cu(1)					129.5(3)
N(2)-N(3)-Cu(1)					124.8(3)
C(10)-N(4)-Cu(1)					111.4(3)
C(1)-S(1)-Cu(1)					95.55(15)
N(3)-Cu(1)-N(4)					80.92(15)
N(3)-Cu(1)-Cl(1)					173.26(12)
N(4)-Cu(1)-Cl(1)					97.45(11)
N(3)-Cu(1)-S(1)					83.38(11)
N(4)-Cu(1)-S(1)					163.77(11)
N(3)-Cu(1)-N(4)					80.92(15)

Table S2. Hydrogen Bond Distances (Å) and Angles (deg) for **HL[2], HL[3], HL[5] and C3a**

D–H···A	d(H···A)	d(D···A)	∠(DHA)	Symmetry transformation for acceptor
**HL[2]**				
N(2)-H(2N)···N(4)	2.20	3.043(3)	166.7	-*x*, *y*-1/2, -*z*+1/2
N(1)-H(1N) ···N(3)	2.16	2.626(3)	114.0	*x*, *y*, *z*
**HL[3]**				
N(1A)-H(1NA)···O(1*w*)	2.37	3.202(8)	161.9	*x*, *y*, *z*
N(2A)-H(2NA)···S(1A)	2.55	3.373(5)	161.2	-*x*+2, -*y*, -*z*+1
N(1B)-H(1NB)···O(2*w*)	2.28	3.097(7)	159.7	*x*, *y*, *z*
N(2B)-H(2NB)···S(1B)	2.58	3.436(5)	170.6	-*x*+1, -*y*+2, -*z*+1
O(1*w*)-H(1W1)···N(4A)	2.08(3)	2.940(8)	164(7)	*x*, *y*, *z*
O(2*w*)-H(2*w*2)···N(4B)	2.03(3)	2.893(7)	163(7)	*x*, *y*, *z*
O(2*w*)-H(1*w*2)···O(1*w*)	2.02(2)	2.897(7)	173(7)	*x*, *y*, *z*
**HL[5]**				
N(2)-H(2N)···N(4)	1.93	2.603(2)	133.8	*x*, *y*, *z*
N(1)-H(1N) ···N(3)	2.10	2.574(2)	114.5	*x*, *y*, *z*
**C3a**				
N(1)-H(1N)···O(1D)	2.09	2.922(6)	163.3	*x*-1, *y*, *z*-1
